# Cleaning up while Changing Gears: The Role of Battery Design, Fossil Fuel Power Plants, and Vehicle Policy for Reducing Emissions in the Transition to Electric Vehicles

**DOI:** 10.1021/acs.est.3c07098

**Published:** 2024-02-13

**Authors:** Matthew Bruchon, Zihao Lance Chen, Jeremy Michalek

**Affiliations:** † Department of Engineering & Public Policy, Carnegie Mellon University, Pittsburgh, Pennsylvania 12513, United States; ‡ Department of Engineering & Public Policy, Carnegie Mellon University, Pittsburgh, Pennsylvania 15213, United States; § Department of Mechanical Engineering, Carnegie Mellon University, Pittsburgh, Pennsylvania 15213, United States; ⊥ Department of Civil & Environmental Engineering, Carnegie Mellon University, Pittsburgh, Pennsylvania 15213, United States

**Keywords:** electric vehicles, life cycle assessment, greenhouse gas emissions, criteria air pollutants, externality valuation, Li-ion batteries, power systems, public policy

## Abstract

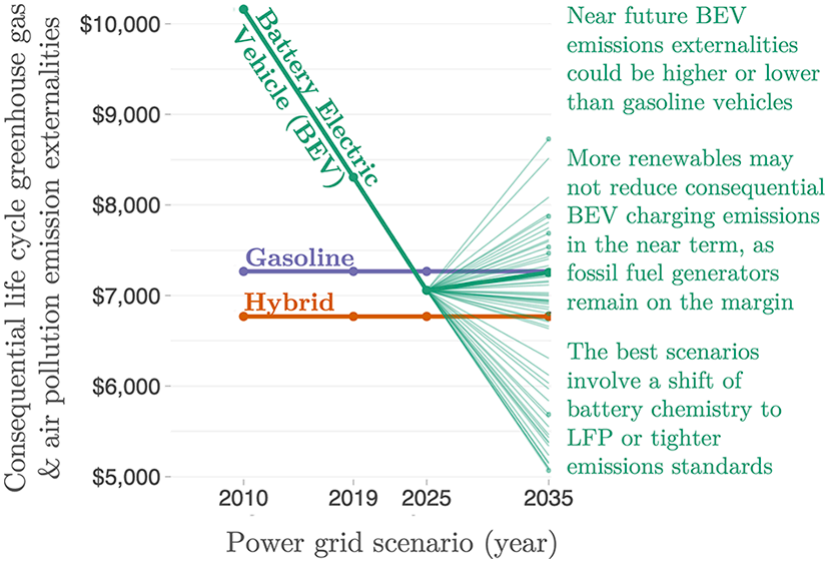

Plug-in electric vehicles (PEVs) can reduce air emissions when charged with clean power, but prior work estimated that in 2010, PEVs produced 2 to 3 times the consequential air emission externalities of gasoline vehicles in PJM (the largest US regional transmission operator, serving 65 million people) due largely to increased generation from coal-fired power plants to charge the vehicles. We investigate how this situation has changed since 2010, where we are now, and what the largest levers are for reducing PEV consequential life cycle emission externalities in the near future. We estimate that PEV emission externalities have dropped by 17% to 18% in PJM as natural gas replaced coal, but they will remain comparable to gasoline vehicle externalities in base case trajectories through at least 2035. Increased wind and solar power capacity is critical to achieving deep decarbonization in the long run, but through 2035 we estimate that it will primarily shift which fossil generators operate on the margin at times when PEVs charge and can even increase consequential PEV charging emissions in the near term. We find that the largest levers for reducing PEV emissions over the next decade are (1) shifting away from nickel-based batteries to lithium iron phosphate, (2) reducing emissions from fossil generators, and (3) revising vehicle fleet emission standards. While our numerical estimates are regionally specific, key findings apply to most power systems today, in which renewable generators typically produce as much output as possible, regardless of the load, while dispatchable fossil fuel generators respond to the changes in load.

## Abstract 2

We find that reducing consequential life cycle air emission externalities over the next decade of transition to electric vehicles depends more centrally on the battery design, fossil fuel power plant emission, and vehicle emission policies than renewable generation capacity.

## 1 Background

**Figure 1:**
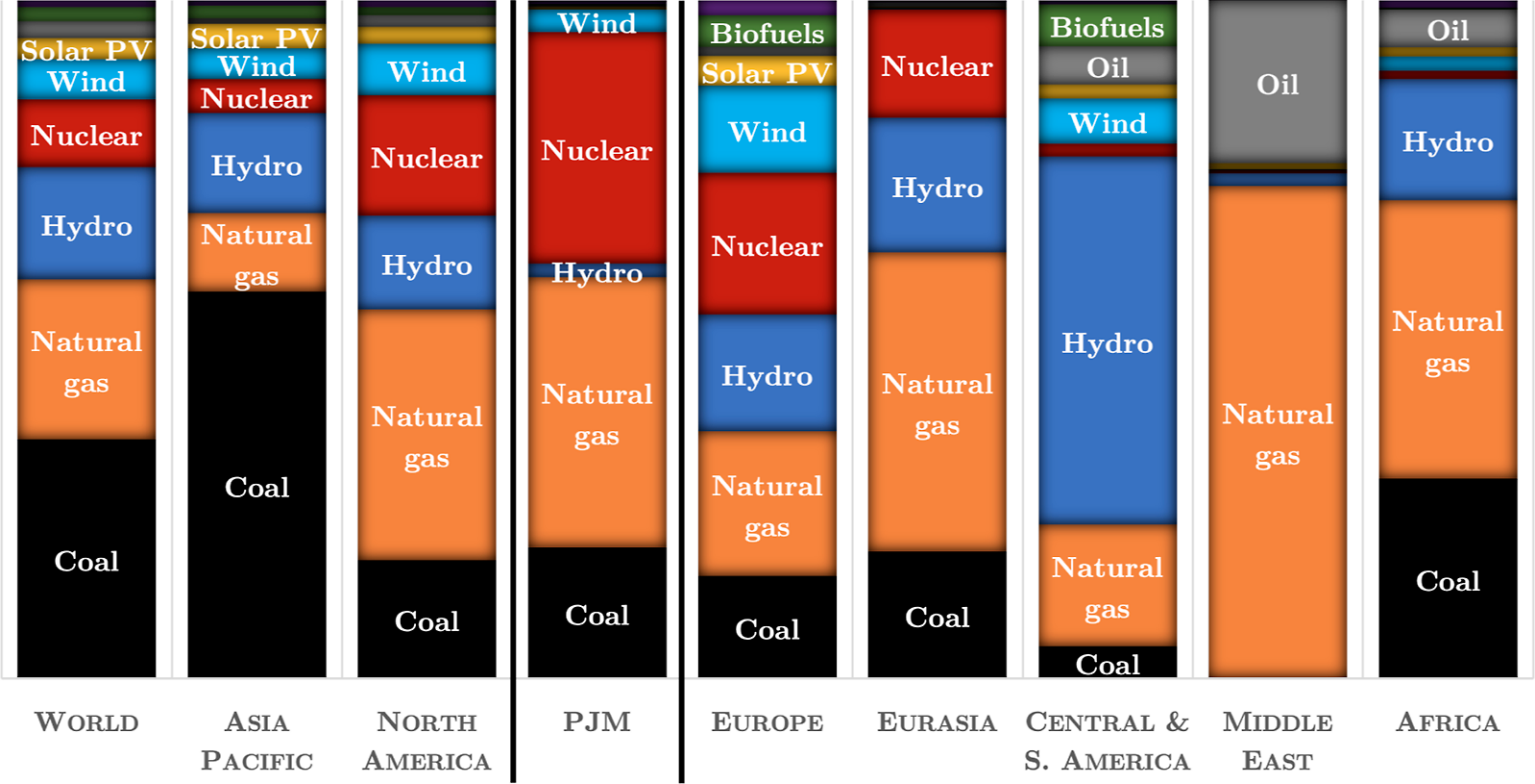
Electricity generation mix by region in 2020. Regions other than PJM are ordered from the largest to smallest total regional generation. Generator fuel types are ordered (bottom to top) from the largest to smallest global generation. Generally, PJM has similar properties to most other regions of the world: wind, solar, and nuclear power produce a minority of generation with low marginal cost, typically generating as much energy as possible regardless of the variations in load, while dispatchable fossil fuel plants (primarily coal and natural gas) adjust the generation in response to changes in load. Hydroelectric generation, a small source in PJM, can adjust the timing of generation within constraints (such as lake level limits) (Data from refs (1) and (2)).

Plug-in electric vehicles (PEVs) offer potential for helping to mitigate climate change and reduce health damages from air pollution when charged with clean power sources. However, electric vehicles charged with coal-generated power can potentially increase greenhouse gas (GHG) and criteria air pollutant (CAP) emissions relative to gasoline vehicles.^19,27,42,49^ The PJM Interconnection is the largest and one of the most coal-heavy regional transmission organizations (RTOs) in the US, coordinating the movement of electricity through all or parts of Delaware, Illinois, Indiana, Kentucky, Maryland, Michigan, New Jersey, North Carolina, Ohio, Pennsylvania, Tennessee, Virginia, West Virginia, and the District of Columbia and serving a customer base of 65 million people. The generation mix in PJM is similar to that of North America as a whole (Figure 1). In 2016, Weis et al. estimated the externalities of PEV consequential life cycle air emissions within PJM, that is, the unpriced costs of emissions arising as a consequence of PEV adoption and use across their life cycle, including changes in grid emissions induced by the additional electricity load from PEV battery charging. Using PJM electric grid data from 2010, their study estimated that if 10% of the PJM region’s vehicles were switched to battery electric vehicles (BEVs), the consequential life cycle emission externalities would be 2 to 3 times as high as those of conventional gasoline vehicles or gasoline hybrids.^49^ The study predicted that PEV emissions would improve with anticipated coal plant retirement in PJM.

Since the time of the Weis et al. study,^49^ average emissions have fallen across the US power sector. Holland et al. (2020) estimate that nationwide overall electricity grid emission externalities fell from $245 billion to $133 billion from 2010 to 2017, with “composition effects” (e.g., coal generation retirements and gas generation installations) and “technique effects” (e.g., SO_2_ control technologies for coal plants) contributing roughly equally to that decline.^20^ Andaloussi estimates $152 billion in health benefits (as a conservative lower bound) from generation fleet changes from 2005 to 2014, with emission abatement techniques accounting for over 50% of emission reductions and fuel switches and retirements accounting for an additional 20%.^3^

Within PJM, the generation mix has changed dramatically due to the widespread retirement of coal plants and installation of natural gas plants. Table 1 summarizes the change in generation capacity from the study of Weis et al.^49^ to this study. The installed capacity of natural gas increased by 60%, largely in the form of relatively efficient combined-cycle natural gas units, while both coal- and fuel oil-generating capacity fell substantially. Furthermore, echoing the finding of Holland et al. (2020),^20^Table S9 shows that the PJM coal generator fleet’s observed average emission factors for many pollutants fell from 2010 to 2019 (SO_2_ by 76%) due to a mix of improved control technologies and retirements of older, less clean plants.

**Table 1:** Evolution of the PJM Generation Fleet’s Installed Capacity (GW) by Fuel Type from 2010 (Analysis Year of Weis et al.^49^) to 2019 (This Study’s Analysis Year)^a^

	capacity (GW)		
	2010	2019	change
gas	48.5	78.2	+29.7
coal	68.0	56.3	–11.7
wind	4.4	10.6	+6.3
oil	10.2	6.3	–3.9
solar	0.1	2.0	+1.9
nuclear	30.6	32.3	+1.7
hydroelectric	8.0	8.9	+0.9
solid waste	0.7	0.7	+0.0

a Wind and solar values include some units that only participate in energy markets (not capacity markets) and are nameplate capacities, not the derated values used in capacity markets.^28,29^

**Figure 2:**
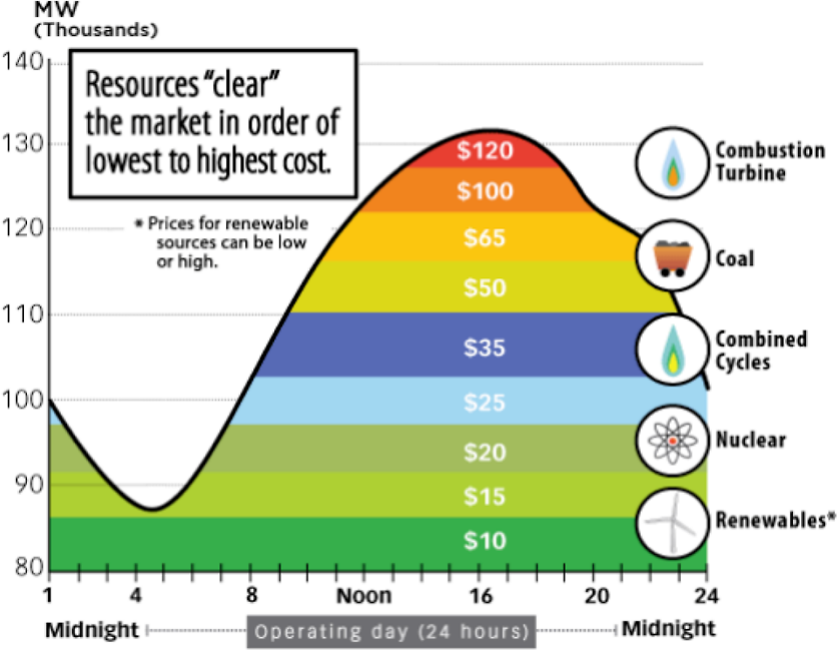
Simplified image used by PJM to explain why the type of generators dispatched to supply electricity at a particular time depend on the total load at that time. A marginal increase in load at a particular time tends to increase generation from the marginal generator type. Additional factors beyond cost, not shown here, can also affect dispatch order (Reprinted with permission from ref (54). Copyright 1999−2024 PJM).

Though this evolution has substantially reduced *average* emissions per unit energy from the PJM grid, it is not immediately clear how it has affected the *changes* in grid emissions induced by PEV charging. A change in PEV charging load (created, for example, by replacing a gasoline vehicle with an electric vehicle) can induce increases in generation from different kinds of generators with different emission profiles depending on the overall level of demand and other factors that vary throughout the day and across seasons (Figure 2). The consequential change in grid emissions induced by PEV use is the relevant quantity for understanding how changes in PEV adoption will affect net emissions, and it is the relevant quantity for understanding the emission implications of PEV policy.^43^ We update the Weis et al.^49^ study, applying its optimization-based consequential power grid emission estimation approach to updated data reflecting the state of PJM in 2019—the most recent year for which all needed data are available. This allows us to investigate a question of policy relevance: To what degree has PJM’s rapid shift in generation technology mix improved PEV consequential life cycle emission externality costs relative to conventional vehicles, and what factors have the most influence in determining future consequential life cycle emissions from PEVs?

The numerical estimates in our study are specific to the PJM region of the United States; however, globally, most power systems today have similar properties (Figure 1), where renewable generators make up a minority of total generation and operate as must-take, generating as much energy as possible regardless of demand, rarely curtailing generation, and rarely responding to changes in load. Globally, in most power systems today, dispatchable generators––mainly coal and natural gas generators that can adjust the output in response to changes in load––are typically the generators whose emissions change when the demand for electricity increases. Because of this, similar dynamics are at play in most power systems today. For example, Holland et al. (2022) show the decoupling of renewable growth and marginal generator emissions to be consistent across all three U.S. interconnections.^18^

In the long run, if renewable capacity grows to the point where renewable generation is routinely curtailed for exceeding demand and storage capabilities, then renewable generation may become a larger or even a dominant share of consequential emissions from changes in PEV charging. Large, persistent future increases in PEV demand for electricity could also induce new plant construction, including renewable generators, depending on economic and policy conditions.^15^ In the near term, however, the emission consequences of PEV charging will continue to be more dependent on the effects of charging load on fossil generation, so emission rates from fossil generators remain a key factor in understanding PEV life cycle emissions. The US Environmental Protection Agency has proposed new power plant standards that, if enacted, would tighten fossil fuel emission limits and address exactly this issue.^46^

In addition to power sector emissions, we also find that battery chemistry, especially the use of nickel, has notable implications for air emission implications of battery production, and the current shift away from nickel-based chemistries toward lithium iron phosphate (LFP) chemistries could have substantial benefits for EV life cycle implications.

We also find, consistent with prior literature, that because US federal light-duty vehicle standards effectively cap fleet-wide emissions, EV adoption does not necessarily reduce net vehicle fleet emissions and, because of the design of the standards, can actually trigger increases in total permitted fleet emissions instead. Thus, the design of fleet standards is a key factor determining the emission consequences of EV adoption over the next decade. The US Environmental Protection Agency has proposed stringent new light-duty vehicle fleet greenhouse gas emission rules that are estimated to effectively require automakers to electrify two-thirds of their vehicle fleet by 2032.^45^ We discuss the implications of this proposed policy.

### 1.1 Literature

To a large extent, conclusions regarding PEV air emissions relative to other powertrains may depend on whether the assessment is attributional or consequential, how power grid emissions are estimated, which life cycle stages are considered, and which pollutants are included. One meta-analysis specific to grid emissions considered results across 32 power grid emission models and found that model estimates can deviate from mean model results by as much as 68% depending on choices such as whether emission factors represent average load or a change in load.^37^Table 2 summarizes relevant studies since this study’s predecessor, Weis et al.,^49^ that compare life cycle emissions of PEVs to those of other vehicles.

**Table 2:** Selection of Relevant Works Comparing Life Cycle Emissions of PEVs to Non-PEVs Since Weis et al. (2016)^a^

study	life cycle assessment characteristics				powertrains	broad findings regarding US PEV emissions
	type	all stages	pollutants	valuation		
Elgowainy et al. 2018^11^	A	yes	GHG	no	12 types	attributed GHGs for PEV < ICEV
Desai et al. 2019^8^	A	yes	GHG	no	I, H, PH, B	attributed GHGs and cost savings vary with annual VMT and regional grid
Kawamoto et al. 2019^25^	A	yes	GHG	no	I, B	attributed GHGs lower for BEV than ICEV when lifetime VMT is above $38K
Woody et al. 2022^52^	A	yes	GHG	no	I, B	100% BEV postal truck fleet attributed 57% lower emissions than a 90% ICEV fleet
						
Holland et al. 2016^19^	CM	no	GHG, CAP	yes	I, B	BEV marginal externalities < ICEV in West, BEV > ICEV in East on average
Gai et al. 2019^16^	CM	yes	GHG	no	I, B	BEV ≪ ICEV in Ontario
Holland et al. 2020^20^	CM	no	GHG, CAP	yes	I, B	BEV ≪ ICEV in West, < ICEV in East on average
Tong et al. 2020^44^	CM	yes	GHG, CAP	yes	I, H, B, NG	HEV < BEV in much of East and Midwest
Holland et al. 2022^18^	CM	no	GHG	yes	I, B	increasing marginal emission factors will offset >50% of BEV GHG reductions
						
Nopmongcol et al. 2017^33^	CS	no	CAP	no	I, H, PH, B	electrification of light-duty vehicles slightly lowers use-phase PM_2.5_ in some regions
Sheppard et al. 2021^40^	CS	yes	GHG	no	I, B	BEV causes 46–49% lower GHGs than ICEV, depending on the charging scheme
Jenn et al. 2023^21^	CS	yes	GHG	no	I, B	BEV causes lower GHGs than ICEV in CA
this study	CS	yes	GHG, CAP	yes	I, H, PH, B	PEV consequential emissions ↓ 17% 2010–2019, but still BEV > HEV and ICEV

a Study types: A = attributional, CM = consequential via marginal factors, and CS = consequential via simulation. Pollutants: “GHG” = greenhouse gases, “CAP” = criteria air pollutants. “Valuation”: whether emissions’ external cost valuations are provided. Powertrains: I = conventional internal combustion engine, H = standard gasoline hybrid electric, PH = plug-in hybrid, B = battery electric, NG = natural gas.

A fundamental factor distinguishing studies is whether they use attributional or consequential life cycle assessment (LCA). Attributional LCA studies estimate emissions and allocate them to activities, requiring the modeler to make decisions about which emissions to allocate to which activities, especially in interconnected systems like the power grid, where many generators feed into a network that serves many loads. In contrast, consequential LCA studies estimate how emissions change in response to an action, decision, or activity. Consequential assessments are relevant for understanding how a proposed policy intervention or action will change net emissions.^9,43,47^

#### 1.1.1 Attributional Studies

Four of the studies in Table 2 use attributional LCA and focus only on greenhouse gas (GHG) emissions, estimating that GHG emissions attributed to PEVs are lower than those of conventional internal combustion engine vehicles (ICEVs), given assumptions for vehicle use patterns.^8,11,25,52^ While these attributional studies may provide meaningful descriptions of emissions attributed to PEVs under reasonable assignment schemes, they do not estimate how changes in PEV adoption would affect power system emissions. Consequential modeling is necessary to estimate the emission implications of changes in PEV use and/or adoption and to inform policymaking that may affect PEV use and/or adoption.^43^

#### 1.1.2 Consequential Studies

Consequential LCA studies aim to estimate the net change in emissions or impacts resulting from an action such as replacing ICEVs with PEVs. Such consequences can be wide-ranging, such as (1) reduced gasoline demand triggering lower prices for gasoline on the world market and inducing new demand or (2) increased PEV adoption increasing consumer awareness and triggering new PEV adoption or new legislation. However, efforts to model such consequences typically involve general equilibrium models that model interactions across the economy and rely on assumptions that are difficult to validate, and the LCA literature on PEVs has focused on consequential emissions from the changes in electricity demand due to PEV charging.

Within the recent literature on consequential PEV charging emissions, complicated trade-offs remain between PEVs and ICEVs. One key factor is whether the study uses regression-based marginal emission factors (MEFs) (which estimate observed correlations between historical changes in power grid load and changes in power sector emissions) or simulation-based estimates (which estimate simulated emissions in scenarios with and without PEV charging load).^10,43^

#### 1.1.3 Regression-Based Marginal Charging Emissions

The regression-based MEF studies in Table 2 find that there is substantial geographic variation in emissions induced by PEV charging, and the choice of the charging scheme can substantially change consequential PEV emissions.^13,44^ Holland et al. (2016), using data from 2010 to 2012 and monetizing damages from both GHGs and criteria air pollutants (CAPs), found that consequential use-phase emissions from BEVs were higher than those of ICEVs in much of the eastern US, and an updated analysis using 2017 data found that PEV use-phase emissions had dropped below those of ICEVs in much of the eastern US.^20^ A more recent study by Holland et al. models linear trends in marginal emission factors over time and finds that even as average emission factors have fallen nationwide, marginal factors have risen enough to offset over half of the potential GHG reductions from the reduced ICEV use.^18^ All of the studies by Holland et al., however, focus on use-phase emissions and do not estimate full life cycle emissions. Gai et al.^16^ focus on GHG emissions only, finding that consequential GHG emissions are lower for BEVs than ICEVs in Ontario, and Tong et al.,^44^ capturing vehicle production emissions and monetizing both GHGs and CAPs, estimate that gasoline hybrids have lower emission externalities than BEVs in most of much of the midwest and eastern US.

#### 1.1.4 Simulation-Based Consequential Charging Emissions

Regression-based MEFs are useful for historical lookbacks or to assess how small changes may have affected a recent grid, but simulation is suitable to assess large-scale changes and future grid scenarios.^10,43^ Future grid mix is a key factor in assessing the consequential emissions of large-scale vehicle fleet transitions since most vehicles purchased today will still be in use years from now when the grid will have evolved.

Within the simulation-based consequential literature, recent studies are limited by their scope of life cycle assessment stages or pollutants. Nopmongcol et al. find that use-phase consequential emissions are lower for each tested PEV than for ICEVs, but it does not consider GHGs and excludes vehicle and battery manufacture and disposal.^33^ Sheppard et al.^40^ find that life cycle consequential emissions are 49% lower for BEVs than for ICEVs, and Jenn^21^ similarly finds that BEVs have substantially lower consequential GHG emissions than ICEVs in California, but both studies exclude CAPs such as nitrogen oxides (NO_*X*_) and sulfur dioxide (SO_2_).

Evidence suggests that excluding certain life cycle stages or pollutants can alter results. Emission costs from the material supply chain, battery production, and automobile manufacturing stages are often substantively higher for PEVs, and excluding those stages from an analysis can underestimate the relative emission costs of PEVs or even change the direction of the result. Similarly, excluding certain CAPs may qualitatively change emission comparisons. For example, excluding SO_2_ (a large share of emission costs from coal plants without advanced abatement technologies and from battery manufacture) may underestimate the relative costs of PEVs. This may help explain why all studies in Table 2 that exclude CAPs are relatively favorable to PEVs, while all those including both CAPs and GHGs––echoing older studies including Weis et al. and Babaee et al. (2014)—find the answer is more pessimistic toward PEVs or lacks a consistent trend.^5,49^

Other literature has compared consequential emissions across different management strategies for PEVs. Jenn et al. (2020) include only use-phase emissions because their goal is to compare business as usual charging to extreme fast charging (which finds often results in higher emissions); Saleh et al. (2022) consider life cycle emissions with different levels of vehicle sharing (which affects fleet vehicle turnover rates) and find that if charging is optimized for GHGs, life cycle GHGs can be reduced up to 50%.^24,38^ Additionally, recent work has suggested that persistent increases in power system load can affect the economics of renewable capacity expansion, triggering the construction of new generators, depending on economic and policy conditions.^15^ We do not assess the potential for PEV-induced capacity expansion in this study.

### 1.2 Contribution to the Literature

From the time since Weis et al.^49^ found that some PEVs have consequential emissions 2 to 3 times higher than ICEVs in PJM, no other studies (to our knowledge) have conducted a consequential assessment of future life cycle GHG and CAP emissions across powertrains. It is unclear whether the studies in Table 2 would have found different results if they had done so. Our study aims to understand how consequential PEV emissions have changed and where trends are heading while identifying key factors that will mostly affect near-future PEV consequential life cycle implications.

We leverage the method of Weis et al.,^49^ which allows us to investigate whether a large rapid transition to PEVs in PJM (10% of all personal vehicles replaced entirely by either new BEVs or new plug-in hybrid electric vehicles (PHEVs)) would increase or decrease the total life cycle consequential emission externalities in both recent and future scenarios, and we conduct sensitivity analysis to identify the key factors determining the direction and magnitude of this result. We consider the vehicle life cycle from mining through vehicle disposal/recycle and the fuel life cycle from feedstock development to combustion. In addition to GHGs, we consider key CAPs, including SO_2_, NO_*X*_, fine particulate matter (PM_2.5_), volatile organic compounds (VOC), and carbon monoxide (CO).

## 2 Results

We present our results by (1) summarizing the evolution of consequential PEV life cycle air emission externalities over time; (2) assessing three levers with the potential to lower near-term consequential emissions; and (3) assessing the sensitivity to variation in the fleet of generators, charging patterns, and the social cost of carbon. An online dashboard summarizing our results and allowing the user to dynamically change scenarios can be found at https://mbruchon.shinyapps.io/PJM_EV/.

### 2.1 Evolution of Consequential PEV Emissions over Time

**Figure 3:**
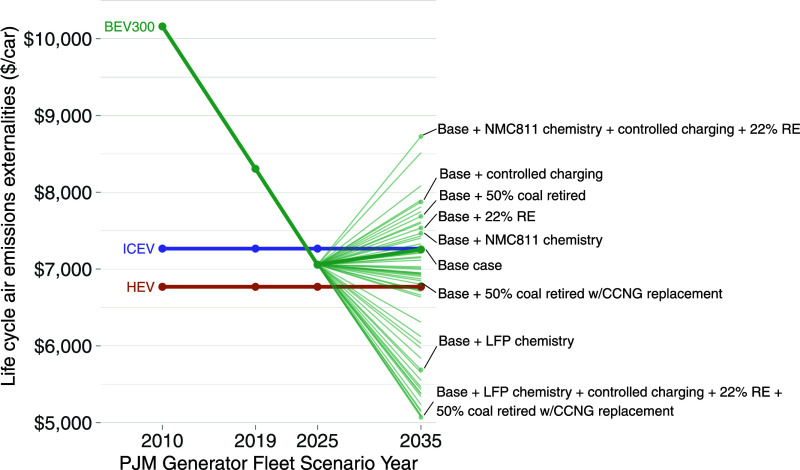
Estimated consequential life cycle emission externalities as PJM’s generator fleet evolves over time for several powertrains when 10% of the light-duty passenger car fleet in PJM’s service area is replaced with new cars of several powertrain types. Each 2035 scenario is shown as a line, including all combinations of renewable penetration (10 or 22%), additional coal retirement (0, 25, and 50%), coal replacement (none or natural gas), battery chemistry (LFP, NMC622, and NMC881) and charging scheme (uncontrolled or controlled) scenarios. Only the two extreme cases and each univariate sensitivity case are labeled for readability. A list of all scenarios is provided in the Supporting Information. For years 2010, 2019, and 2025, only the base case scenario is shown (for readability). “ICEV” = conventional internal combustion engine vehicle, “HEV” = gasoline hybrid electric vehicle, “BEV300” = battery electric with a battery range of 300 miles, “LFP” = lithium iron phosphate BEV battery chemistry, and “NMC” = nickel manganese cobalt lithium-ion BEV battery chemistry. For BEV300, the base case includes uncontrolled charging, NMC622 battery chemistry, 10% renewables in 2035, and no accelerated coal retirements or natural gas installations. The *y*-axis is truncated to make the trends more visible.

**Figure 4:**
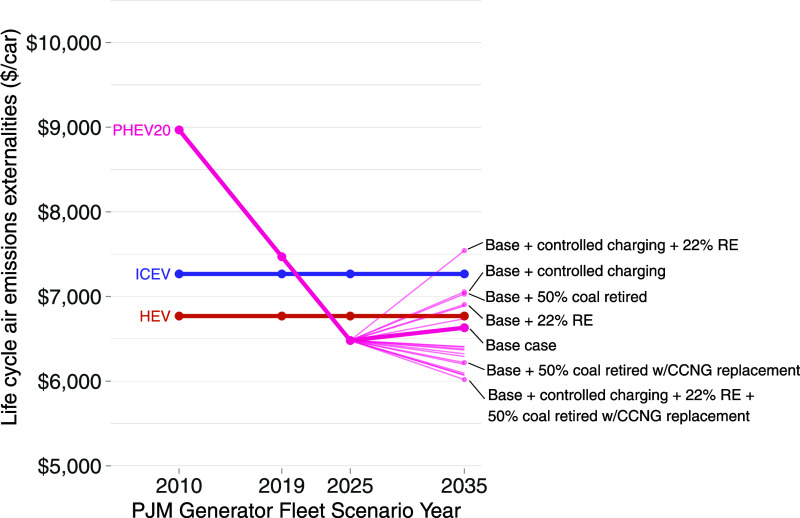
Estimated consequential life cycle air emission externalities as PJM’s generator fleet evolves over time for several powertrains when 10% of the light-duty passenger car fleet in PJM’s service area is replaced with new cars of several powertrain types. Each 2035 scenario is shown as a line including all combinations of 2035 scenarios tested including renewable penetration (10% or 22%), additional coal retirement (0%, 25%, and 50%), coal replacement (none or natural gas), battery chemistry (LFP and NMC111), and charging scheme (uncontrolled or controlled). Only the two extreme cases and each univariate sensitivity cases are labeled for readability. A list of all scenarios is provided in the Supporting Information. For years 2010, 2019, and 2025, only the base case scenario is shown (for readability). “ICEV” = conventional internal combustion engine vehicle, “HEV” = gasoline hybrid electric vehicle, “PHEV20” = plug-in hybrid electric vehicle with a battery range of 20 miles. For PHEV20, the base case includes uncontrolled charging, NMC111 battery chemistry, 10% renewables in 2035, and no accelerated coal retirements or natural gas installations. The *y*-axis is truncated to make trends more visible.

Figures 3 and 4 illustrate the evolution of the estimated consequential life cycle emissions for BEVs and PHEVs, respectively, over time in PJM. Relative to the 2010 generator fleet, the 2019 generator fleet results in consequential life cycle emissions that are 18% lower for BEVs and 17% lower for plug-in hybrid electric vehicles (PHEVs). However, this substantial decline from 2010to 2019 does not bring the externality estimates of PHEVs or BEVs below ICEVs or gasoline hybrid electric vehicles that do not plug in (HEVs). HEVs remain the powertrain with the lowest consequential life cycle emission externalities, with an estimate around $6800 over the vehicle’s life cycle. By comparison, externality estimates are 7% higher for ICEVs, 10% higher for PHEVs, and 23% higher for BEVs. Due in part to the lack of reliable future emission projections for other life cycle stages, we only vary over time the inputs pertaining to electricity system operations, so our estimates of ICEV and HEV consequential emissions do not change over time.

The trend of falling consequential life cycle PHEV and BEV air emissions continues through our 2025 scenario at a similar rate. This nearly linear continuation suggests that retirements and installations scheduled through 2025 continue the operational trends observed from 2010 to 2019. In 2025, PHEV consequential emissions ($6500) are valued just below those of HEVs ($6800), but BEV consequential emissions ($7100) remain 20% larger than PHEVs and slightly higher than HEVs.

**Figure 5:**
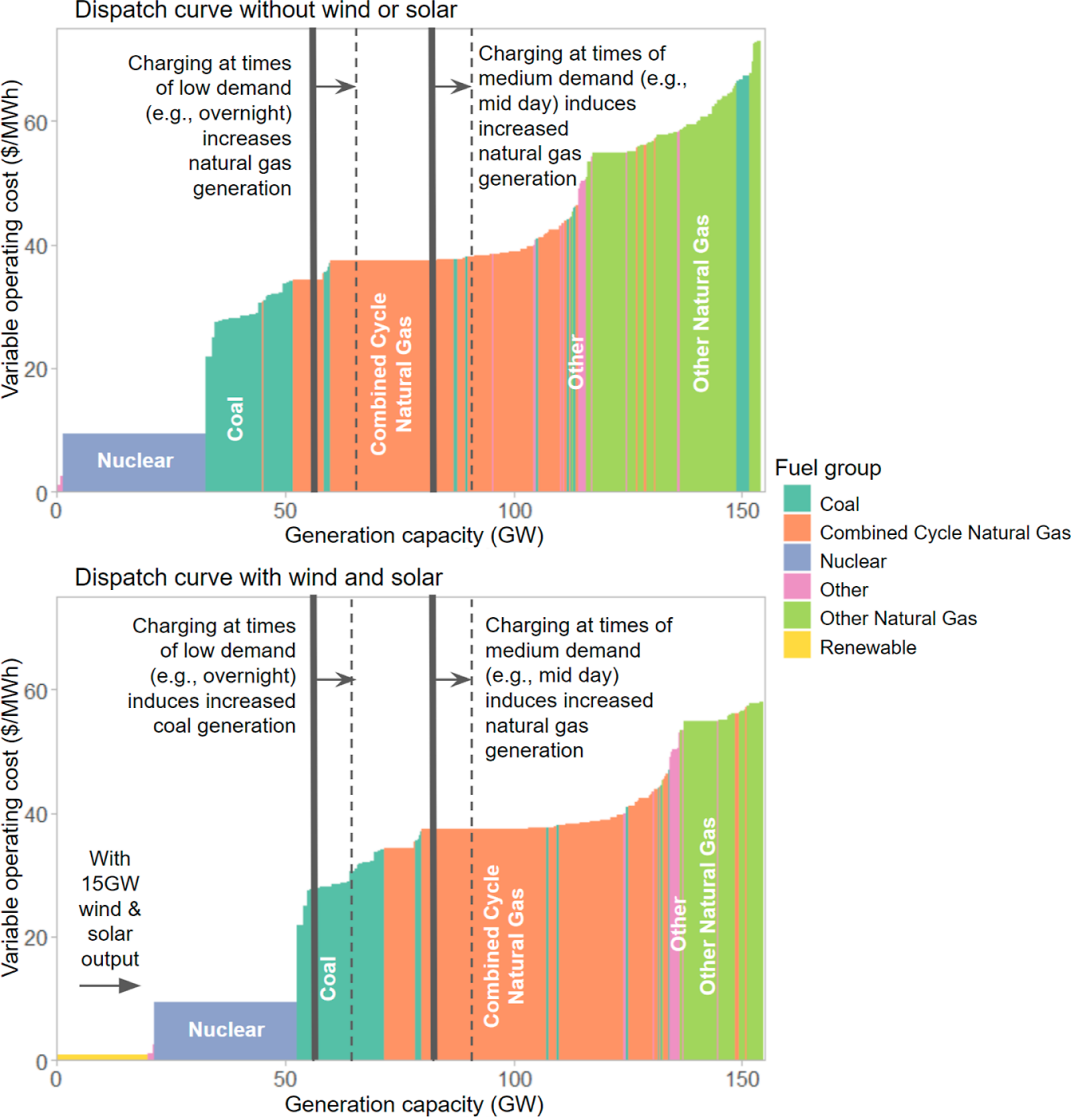
Illustrative example dispatch curves for our 2035 scenario showing how wind and solar generation shift the dispatch curve and can increase the presence of coal on the margin at times that PEVs charge, even while decreasing overall grid emissions. Median demand in our 2035 scenario is around 80 GW. The size of the shift is exaggerated for purposes of illustration. Actual dispatch depends on more factors than the cost-ordered dispatch curve shown here, and Figure S11 shows precise shifts in generation for the 2035 scenario.

In our base case 2035 scenario, the downward trend in PEV emissions reverses. Even though average grid emissions drop as renewable penetration increases, consequential emission externalities from PEV charging increase somewhat because additional renewable generation shifts the dispatch order and changes in which fossil fuel plants respond to changes in load, resulting in more coal on the margin. Figure 5 provides an illustration showing how renewable capacity expansion may increase or decrease the degree to which coal operates on the margin at the times PEVs charge. While the dispatch order curves provide a good sense of the issue, the precise shift in generation induced by PEV charging depends on factors beyond the dispatch order, such as constraints on ramp rates, reserves, and generation, and they depend on the distribution of times that PEVs charge throughout the day and year. The actual portion of each generator type induced by PEV charging for our 2035 base case for cases of uncontrolled charging (charging upon completion of the last trip of the day) and controlled charging (charging at times of the lowest cost) is summarized in Figure S11. In the 10% renewable base case, BEV externality valuations are on par with those of ICEVs, while PHEVs and HEV are similar (both lower than those of BEVs and ICEVs). In the 22% renewable scenario (PJM’s own “Policy” case), HEVs are once again the lowest emitting powertrain, and life cycle consequential emissions from BEVs are 11% larger. Thus, increasing the renewable capacity alone is not sufficient in the near term to reduce consequential emissions from PEV charging, and increased renewable capacity can actually increase consequential PEV charging emissions in the near term.

### 2.2 Three Key Variables Impacting Consequential Air Emissions

**Figure 6:**
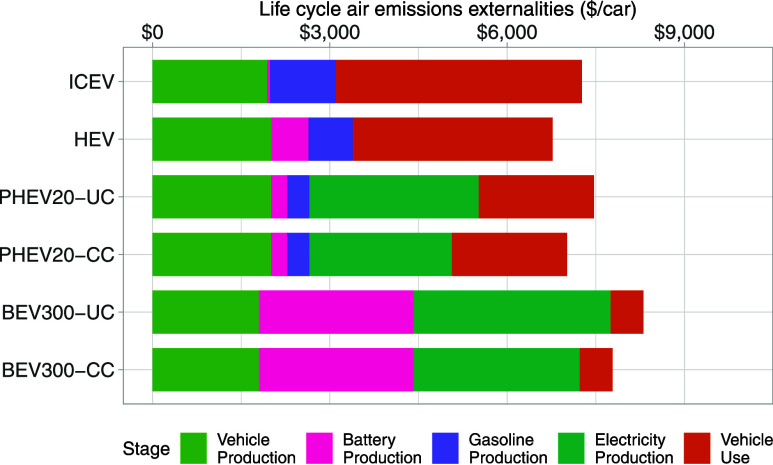
Consequential life cycle air emission externalities per vehicle in 2019, assuming 10% of the light-duty passenger car fleet in PJM’s service area is replaced with PEVs. “ICEV” denotes a conventional internal combustion engine vehicle, “HEV” denotes a standard gasoline hybrid electric vehicle (NiMH battery), “PHEV20” denotes a plug-in hybrid electric vehicle with a battery range of 20 miles (Li-ion battery with NMC111 cathode chemistry), and “BEV300” denotes a battery electric with a battery range of 300 miles (Li-ion battery with NMC622 cathode chemistry). “CC” indicates that battery charge schedules are optimally controlled by PJM to minimize system operation costs, and “UC” indicates that battery charging is uncontrolled (i.e., initiated by the vehicle owner as soon as they complete their daily driving and arrive home. “Production” includes disposal and recycling; “Vehicle Use” includes tailpipe emissions and tire and brake wear).

Figure 6, which breaks out air emission externality valuations by life cycle stage, highlights that battery manufacture and electricity generation are the major sources of PEV externalities in our 2019 base case. Synthesizing results across sensitivity tests, Figures 3 and 4 show that these two emission sources are reduced in a consistent manner by two variables: battery chemistry and the fossil fuel generator fleet mix. We assess each of these in turn and also separately assess a third variable ignored in our primary cases but identified as important in prior literature: policy interactions with federal fleet standards.

#### 2.2.1 Battery Production

**Figure 7:**
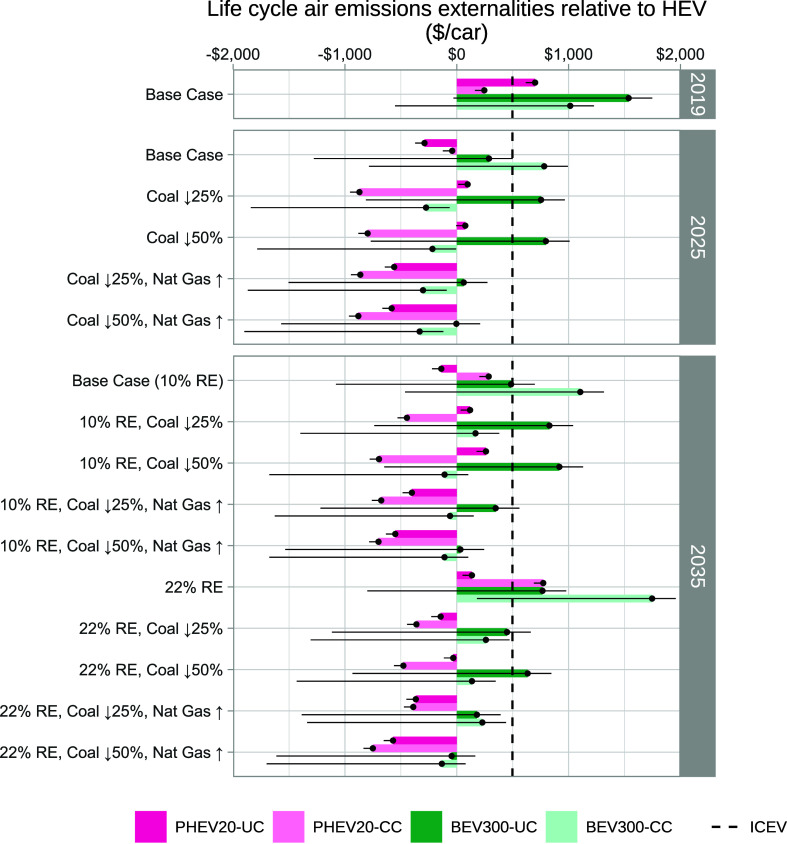
Range of life cycle emission externalities relative to an HEV ($0 mark on *x*-axis) and ICEV (dashed line) across sensitivity cases. “PHEV20” denotes a plug-in hybrid electric vehicle with a battery range of 20 miles, and “BEV300” denotes a battery electric with a battery range of 300 miles; “UC” and “CC” indicate uncontrolled and controlled charging schemes, respectively. Horizontal error bars show the range of values across battery chemistries. For both PHEVs and BEVs, LFP is the lowest-externality chemistry. For PHEVs, NMC111 is the highest (and the base case). For BEVs, NMC622 is the base case and NMC811 is the highest.

One key uncertainty for PEVs is battery chemistry. Shifting the lithium-ion battery chemistry from nickel manganese cobalt (NMC) to LFP is the most impactful single factor lowering consequential life cycle emissions of PEVs, as shown in Figure 3. In each power grid scenario we run (Figure 7), we also assess a set of several potential battery chemistries. Using all other 2019 base case assumptions but switching BEVs from the NMC622 chemistry to LFP lowers battery production externalities from around $2600 to around $1000 (this altered breakdown by life cycle stage is shown in Figure S7.) In every scenario, LFP, the lowest-externality battery chemistry (leftmost end of the horizontal “error bar” on each scenario), leads to a large reduction in BEV externalities and consistently brings them substantially below those of HEVs.

This shift is largely attributable to the avoided SO_2_ emissions from nickel mining. In our base case (NMC chemistry), battery manufacturing for BEVs has a substantial impact on SO_2_ and also produces meaningful amounts of NO_*X*_ and PM_2.5_ (Figures S3 and S5). (We break down results by the pollutant and life cycle stage in terms of externality valuation in Figures S3 and S4 and Tables S1 and S4 and do so for tonnes of emissions in Figures S5 and S6 and Tables S5–S8.) In monetary valuation terms (using damage estimates for US locations), these criteria pollutant emissions more than offset the higher GHG emissions of ICEVs. LFP batteries drastically reduce these material extraction emissions. We discuss assumptions and implications in more detail in Section 3.

#### 2.2.2 Fossil Fuel Generator Fleet Mix

The detailed consequential emission inventories shown in Figures S3 and S5 show that the PEV consequential grid emissions tend to result in substantial GHGs, NOx, and PM2.5 from combustion plants, even in high-renewable scenarios.

One option to reduce the fossil fuel generator fleet’s emissions is to accelerate coal retirement. Figure 7 shows how results vary across scenarios for PJM’s generator fleet (described in Methods and Materials), using HEV life cycle air emission externalities as a constant baseline across all grid scenarios. In the 2025 and 2035 accelerated coal retirement cases, PHEVs have lower externalities than ICEVs or HEVs. To drastically reduce BEV consequential emissions under accelerated coal retirement, it is further necessary to ensure that the new generator merit order does not favor relatively inefficient generating units designed to handle peak load, such as open-cycle gas turbines or diesel generators. One pathway shown in Figure 7 is to replace retired coal plants with relatively efficient natural gas-combined cycle generation. An alternative pathway under accelerated coal retirement––though not consistently emission-reducing in other grid scenarios––is for the grid operator to centrally schedule the timing of PEV charging (within vehicle constraints) for cost minimization (“BEV300-CC” option).

#### 2.2.3 Policy Interactions

Prior work finds that policy interactions exist between PEV adoption and federal regulations of light-duty fleet fuel economy and greenhouse gas emission rates. PEV sales relax an automaker’s fleetwide emission targets, triggering higher overall permitted fleetwide emissions.^23^ We describe the dynamics of these policy interactions further in the Supporting Information.

Our base case results do not include these policy interactions, but we analytically assess their impact using the formulation described by Jenn et al. (2019).^22^ We estimate that including those policy interactions adds around $4100 to each PEV’s estimated consequential externalities (Figure S12), raising them above HEVs or ICEVs across all of our 2025 and 2035 scenarios.

Such policy interactions may change if federal rules change or if fleet emissions drop below the levels mandated by fleet standards driven by other forces such that automakers no longer absorb any slack in permitted emissions. We discuss this in more detail in Section 3.

### 2.3 Additional Sensitivity Cases

In addition to test cases pertaining to the generation fleet’s evolution, battery chemistry, and policy interactions, we also consider the sensitivity of our results to PEV charge strategy and valuation of emissions. These cases are briefly summarized here and discussed in full in the Supporting Iinformation.

#### 2.3.1 Minimum Cost Controlled Charging

Prior work demonstrates that when charging is optimally controlled to minimize operating costs, the effect on emissions varies.^50^ In our 2019 scenario, this style of controlled charging reduces externalities for BEVs and PHEVs enough that PHEVs become more favorable to ICEVs (Figure 6). Within the fleet of natural gas generators, controlled charging of BEVs favors relatively efficient, lower-emission units (Figure S1). However, in 2035, controlled charging can increase or decrease consequential emissions depending on the scenario (Figures 3 and 7).

#### 2.3.2 Valuation of Considered Air Emissions

Figure S2 separates the trends over time for CAPs (valued using estimated health damages) and GHGs (valued using climate change impacts). Top-level findings are altered if only one type of emission or the other type of emission is included as an externality cost component. If only CAPs are considered, then BEVs are always the highest-externality option by a substantial margin, and the other three powertrains are similar to one another (with ICEVs sometimes being the lowest). However, the GHG externalities of BEVs are lower than other powertrains (except in the 2019 scenario, when HEVs are slightly lower), consistent with the studies in Table 2 that examine only GHGs.

Recognizing there is little consensus on what value should be assigned to the social cost of carbon,^12,17,30,34,36^ we also consider the value of $185/tonne CO_2_ estimated by Rennert et al.^36^ This value generally raises GHG externalities above CAPs (Figure S13) and shifts the overall comparisons to BEVs (Figure S14). Total externalities rise across the board for all powertrains––from roughly $7000 to $14,000 for ICEVs––potentially increasing the justification for policies to address emissions. We also offer the social cost of carbon as a user-adjustable parameter in our online dashboard^a^.

## 3 Discussion

We find that PEV consequential life cycle air emissions have fallen substantially since 2010 and are likely to continue through at least 2025, but that the trend through 2035 could reverse, depending on the factors including battery chemistry and fossil fuel power generation fleet. Our 2019 grid scenario is somewhat comparable to the future 2018 grid test case in the study by Weis et al. (2016), which used PHEV-35 (this study uses GREET’s latest default PHEV, a PHEV-20, instead).^49^ The study projected that PHEV emissions would be slightly below ICEVs and HEVs by 2018; in contrast, we find that as of 2019 they still remained slightly above both. Another relevant comparison point is from the study of Tong and Azevedo (2020), which used marginal emission factors and found that HEVs were the lowest-externality powertrain choice across the PJM service territory (as of the 2014 grid).^44^

Renewable generators, such as wind and solar power, reduce grid emissions by displacing fossil fuel generation. There is a widespread belief that as renewables are added to the power grid, electric vehicles will therefore become cleaner. This may be the case in the long run if renewable capacity reaches levels where renewables are routinely curtailed and electric vehicle charging can absorb renewable energy that would otherwise be lost. For the near term, however, our results show that the largest lever for lowering consequential life cycle electric vehicle emissions is not increasing wind and solar capacity (which can, counterintuitively, even increase the consequential electric vehicle charging emissions in the near term). Instead, we identify three key levers: (1) reducing emissions from battery production by shifting from nickel- and cobalt-based chemistries to LFP (and/or potentially reducing the battery capacity), (2) reducing emissions from the fossil fuel generator fleet by accelerating coal retirement, and (3) reducing emissions induced by policy interactions by phasing out the features of vehicle fleet standards that increase permitted fleetwide emissions when PEVs are sold. We discuss each lever further here.

### 3.1 Reducing Emissions from Battery Production

The outsize contribution of battery manufacturing and related material extraction processes to externality estimates suggests that battery chemistry and sizing of batteries may warrant more attention.

Nickel extraction and its SO_2_ emissions are particularly outsized contributors to the externalities in our results (and they explain why a switch to LFP brings BEV emissions below other powertrains in our 2019 base case). Most nickel used in NMC lithium-ion batteries currently comes from sulfide ores, the refining of which results in SO_2_ emissions (a smaller portion comes from laterite ore, which does not have sulfur or release SO_2_).^51^ The literature suggests substantial variation across countries in whether SO_2_ emissions are controlled, used to create sulfuric acid, or emitted freely into the atmosphere;^7^ Canada and China have stronger pollution controls relative to other nickel-producing countries, such as Russia.^51^

We use the GREET 2021 model’s default assumptions, which hold battery manufacturing emissions constant over time and are based on a mix of 10 countries in which nickel is extracted (as noted in Limitations, we do not consider any potential differences in the value of reduced mortality risk across countries). It is conceivable that enhanced controls internationally, or a shift toward greater domestic battery-related extraction activity, could reduce the emissions from battery production substantially. In our 2019 base case, reducing battery production emission externalities by roughly 40% would suffice to bring BEVs to parity with HEVs. Given that these supply chain choices alone may swing the answer to whether PEVs have lower externalities than other powertrains, they warrant attention by policymakers, regulators, and manufacturers.

Additionally, we modeled a BEV with a range of 300 miles in our base case, but the average vehicle in our NHTS data sample is driven around 30 miles per day. Shorter range vehicles require fewer batteries, which reduce production emissions. Short of reducing vehicle range, there may also be value in right-sizing vehicles as a pathway to right-size batteries, since larger, heavier vehicles require larger batteries to achieve the same range, while also creating additional safety and emission externalities because of their size and weight.^39^

### 3.2 Reducing Emissions from the Fossil Fuel Generator Fleet

We estimate a substantial 17–18% drop in PEV consequential emission externalities from 2010 to 2019 that will continue steadily through 2025 but not enough close to the gap with ICEVs or HEVs (except potentially for PHEVs). This reinforces the high-level finding of Holland et al. (2022) that falling average GHG emissions have not necessarily resulted in similarly large drops in marginal or consequential GHG emissions from PEV charging.^18^ Our future high-renewable scenarios similarly demonstrate that even as a large proportion of fossil fuel generation is offset, reducing average power grid emissions dramatically, consequential PEV emissions may not necessarily fall until renewable generators represent a large enough portion of the fleet that they are routinely curtailed. The emission rates of generators that operate on the margin at the times electric vehicles charge determines the emission consequences of PEV charging, so emission control standards and coal retirement (both of which were significant from 2010 to 2019, as shown in Table S9) are larger factors affecting consequential PEV emissions.

The deviation in the results for CAPs and GHGs is notable. For policymakers and stakeholders most interested in health impacts and equitable access to clean air, the large emissions of SO_2_ and other CAP emissions from BEVs are concerning, although the rapid decline in BEV CAPs from the grid is heartening, and the potential of LFP chemistries to reduce air pollution is encouraging. For policymakers and stakeholders most concerned about climate change, BEVs appear favorable even if their consequential GHG emissions have not fallen since 2010, and the higher social cost of carbon valuations favor BEVs overall.

### 3.3 Reducing Emissions Induced by Policy Interactions

Because current federal standards increase permitted fleetwide emissions when PEVs are sold, the consequences of PEV adoption in the near term can be to increase net air emission externalities.^23^ Evolution of these policies to phase out leakage is important to ensure that increased PEV adoption does not trigger increased consequential fleet emissions. Additionally, it is possible that the increased adoption of PEVs may trigger consequential changes in the government’s willingness or ability to increase the stringency of fleet standards due to changes in the estimated cost of compliance and its implications for cost–benefit calculations, political considerations, and regulatory authority,^53^ and we do not attempt to model such possibilities here. The framing of this assessment is based on PEV adoption being driven by consumer preferences and nonfederal standards (e.g., California’s ZEV policy or state tax incentives). To the extent that current federal standards themselves have induced growth in the US PEV market––or to which future, potentially increasingly stringent federal standards may become the primary driver of PEV adoption––this framing may become less relevant.^45^

### 3.4 Limitations

This study’s simplifying assumptions should be considered to contextualize results. Our handling of grid dispatch is consequential in nature, but because we do not expect large differences between (1) average vehicle and fossil fuel production emissions and (2) consequential emissions from new production, we follow prior literature in relying on attributional estimates as proxies for consequential estimates for those life cycle stages (GREET is an attributional model). This means that we do not model the potential for large-scale fuel switching to induce changes in each one of the upstream processes that extract and refine those fuels, and we do not model the potential for changes in PEVs to induce changes in supply chain adjustments for battery material processes. Also, while we include sensitivity cases that account for the effects of PEV adoption on vehicle fleet composition under federal fleet emission standards, we do not model the potential for increased EV adoption to induce changes in those standards.^53^

We also do not model the potential for large changes in demand to induce capacity expansion in the power grid. Widespread PEV adoption of the nature modeled in this paper––large enough to have nonmarginal impacts on grid operations––could conceivably have long-term impacts on capacity expansion. Assigning causality between specific changes in the load and specific capacity expansion projects (or emission reductions induced by the average newly installed generation unit) depends on an additional layer of assumptions about economic and policy conditions. Nonetheless, studies in the areas of capacity expansion and cost-minimizing investment have considered this question and often find that the newly induced generating capacity tends to be a relatively low emission, whether due to policy constraints (e.g., California’s Renewable Portfolios Standard) or economic conditions.^21,33,41^ Modeling applying induced capacity expansion to adjust the marginal emission rates suggests that new demand could reduce those rates in the long term, including over PJM’s general geographic region.^14,15,32^

We also exclude the potential for demand shifts to trigger power system policy constraints, trigger political responses, or affect pricing in ways that affect the other sectors of the global economy. Such factors could potentially have other emission consequences that could increase or decrease our estimates.

Further, externality implications of conventional air pollutants depend on the location of release, including the proximity to population centers and variation in the valuation of reduced mortality risk, especially internationally, which raises equity questions. Our estimates are based on locating all supply chain operations in US locations where similar activities occur, so that population exposure and the value of reduced mortality risk are US-based estimates. A range of additional modeling assumptions and limitations is discussed in the Supporting Information.

### 3.5 Implications

Overall, while the long-run ability of vehicle electrification to eliminate emissions from the transportation sector will depend critically on the transition of the power grid to near-zero emission generation sources, like wind and solar power, the near-term emission implications of PEVs depend more critically on battery design (including a shift-away from nickel and cobalt-based chemistries toward LFP), fossil fuel generators (including emission control technology and coal retirement), and public policy design (including a phase out of features in federal fleet standards that increase permitted fleetwide emissions when PEVs are sold as well as policy to reduce fossil fuel generator emissions).^45,46^

We recommend that the policy aiming to encourage PEV adoption as a means to reduce greenhouse gas and traditional air pollution emissions both (1) explicitly model consequential emission externalities to estimate the potential effects of candidate policies and (2) consider complementary policies that may reduce emissions from fossil generators, battery production, and fleetwide emission standards in order to reduce consequential emissions during the transition to electrified transportation. Comparative consequential externality cost estimates, such as the ones we provide here, can help clarify the potential for technology and policy solutions to reduce unpriced social costs and provide guidance informing policy interventions for addressing market failures and encouraging emission reductions.

## 4 Methods and Materials

We estimate PEV consequential emissions as the difference between total life cycle emissions for (1) a “status quo” baseline vehicle fleet and (2) a 90% “status quo”, 10% replacement fleet of the same size, where replacements may be new HEVs, PHEVs, or BEVs. We model scenarios with different power plant fleet specifications, solving each case for minimum cost generation to satisfy load, and we compute the difference in the resulting emissions between our “status quo” and 10% replacement cases to identify consequential emissions. To isolate the impact of generator fleet changes, we hold fixed all other inputs across our base scenarios, including travel patterns, noncharging electricity demand, transfers between PJM and other systems, and transmission constraints.

### 4.1 Optimization Approach

To simulate PJM day-ahead market operations with and without PEV charging loads, we adopt and adapt a unit commitment optimization approach including storage that was formulated by Lueken and Apt (2014).^26^ This approach was subsequently applied to assess PEVs within the context of the New York Independent System Operator (NYISO) and PJM RTOs.^48−50^ As an approximation of the PJM market, the model uses a sliding time window approach similar to model-predictive control, optimizing a 48 h time window, accepting the results for the first 24 h, advancing the optimization window by 24 h, and repeating until a full year is optimized.

The optimization objective is to minimize, across all generators in all transmission constraint regions, the sum of per-MWh variable costs for each generator (including fuel costs), startup costs of bringing each generator online, and fixed costs associated with each generator being online. Supply must match demand minus renewables (which may be curtailed), and transmission constraints across five PJM subregions are included. Additional constraints for each generating unit model their operational characteristics (ramp rate, minimum uptime, and minimum downtime). For grid-scale storage units and PEVs, constraints track their states of charge including charge and discharge efficiency. Storage units can both draw energy and return it to the grid, but PEVs are assumed to only draw energy. The full mathematical formulation is provided in the Supporting Information (Tables S10 and S11).

Several minor changes were made to the prior studies’ optimization code to make it match the specification, due to an implementation issue found during this update. However, running the model with and without this fix suggests that it does not substantially alter results. The full model formulation, code, data, and a user guide are available at https://github.com/mbbruch/PHORUM_EV_2022.

### 4.2 Vehicle and Charging Scenarios

For each powertrain, we model the effects of converting 10% of light-duty passenger vehicles in the PJM service area. We adopt parameters from the GREET model for each powertrain type, including ICEV, HEV (NiMH battery), PHEV (Li-ion battery with NMC111 cathode chemistry) with an all-electric driving range of 20 miles in charge-depleting mode, and BEV (Li-ion battery with NMC622 cathode chemistry) with an all-electric driving range of 300 miles. In order to make charging optimization tractable, PEV charging load is characterized using a weighted set of 15 daily vehicle travel profiles from the 2009 National Household Travel Survey selected to be as representative as possible of the overall fleet patterns, as described by Weis et al. (2014).^48^ We compared 2009 and 2017 National Household Travel Survey (NHTS) data and found that the daily vehicle-distance traveled per household did not change substantially. We consider two alternative schemes for when these aggregated PEV batteries are charged. In one scheme (“uncontrolled charging”), each driving profile begins charging immediately upon arrival at home after the last trip of the day until reaching full charge. In the other scheme (“controlled charging”), the grid operator can choose when and how quickly each driving profile charges at home with the constraint that it must be fully charged before the time its daily travel begins.

### 4.3 Power Grid Data

We update the data sets used by Weis et al.^49^ to be as up to date as possible across data sets at the time of the analysis. This results in a base-modeling year of 2019. The PJM dispatchable generation fleet is characterized primarily from Energy Information Administration (EIA) form 860 data and the Environmental Protection Agency (EPA) National Electric Energy Data System (NEEDS) data set. PJM’s public DataMiner portal provides data on 2019 load, renewable generation, and transfer limits across its transmission interchanges. Fuel price data come from EIA form 923.

### 4.4 Power Grid Scenarios

Our base case scenario is based on the 2019 grid, as described previously. As a near-term future scenario, we model the year 2025, chosen because the volume of planned retirements and installations documented in the EIA-860 forms drops significantly after that year. In addition to those documented plans, we also increase the installed capacity of solar photovoltaic generators and storage to match the 2025 level forecast in a consulting report prepared for the PJM Load Analysis Subcommittee.^6^ For this scenario, we also assume that the current rate of new wind installation will continue through 2025.

To estimate how consequential emissions may be affected by higher levels of wind and solar, we modeled two levels of renewable penetration that are used in a recent PJM analysis framework. PJM’s “Base” 2035 analysis case assumes 10% renewable penetration, and their “Policy” case (consolidating state-level and corporate targets for 2035) assumes 22%.^35^ These higher-renewable scenarios are detailed further in the Supporting Information. Separately from each year’s projected generator retirements and new-generation installations, we also consider for 2025 and 2035 a set of alternative fossil fuel power plant fleet scenarios as sensitivity cases. We describe these scenarios for renewable penetration and generator fleet makeup in the Supporting Information and summarize them in Table S12.

### 4.5 Life Cycle Emission Externality Data

Our life cycle assessment includes: vehicle and battery production (material extraction through vehicle assembly); production (extraction and refining) of fuels for vehicular combustion; electricity generation (including fuel production); vehicle use (including tailpipe combustion, fluids, tire, and brake wear); and vehicle and battery disposal and recycling. Our optimization model determines how much each electricity generating unit creates (megawatt-hours). To quantify each generating unit’s emission factor (lb per megawatt-hour), we use the EPA National Emissions Inventory, filling in any units not found in the Inventory with the average emission factor by NEEDS generator type. Emission factors for all other sources, such as tailpipe combustion and battery production, are taken from the default values within the Greenhouse Gases, Regulated Emissions, and Energy Use in Technologies Model (GREET).^4^ GREET provides attributional life cycle estimates, and we assume that attributional estimates are good proxies for consequential effects for these life cycle stages.

Externality costs from vehicle use, including induced electricity generation, are computed once for the year of purchase and then treated as a recurring annual cash flow that is discounted over the life of the vehicle, using a 3% nominal discount rate (5% real discount rate) and GREET’s assumption of 172,000 miles of vehicle lifetime. This approach does not capture the potential for consequential charging emissions to change over the life of the vehicle.

We aggregate GHG and CAP emissions using a common unit, monetizing climate change and health costs using an Interagency Working Group-recommended social cost of carbon of $51/tonne and the AP3 reduced complexity model to estimate externalities from conventional air pollutants.^31^ We test alternative social cost of carbon values as sensitivity cases. Our approach to valuation of emissions, including simplifying assumptions made to site the locations of production emissions in order to monetize health damages, is described in greater detail in the Supporting Information.

## Data Availability

Data and code supporting the findings of this study and a user guide with detailed information on the model are available at https://github.com/mbbruch/PHORUM_EV_2022. We have created an interactive tool to allow users to adjust model assumptions and see how they affect key results at https://mbruchon.shinyapps.io/PJM_EV.

## Supporting Information Available

Minimum-cost controlled charging; reserve requirements; social cost of carbon; policy interactions; future renewable scenarios; valuation of emissions; and modeling assumptions (PDF)

## Footnotes

a https://mbruchon.shinyapps.io/PJM_EV
