# Supplementary material for: Cleaning up while Changing Gears: The Role of Battery Design, Fossil Fuel Power Plants, and Vehicle Policy for Reducing Emissions in the Transition to Electric Vehicles
Source: Environ Sci Technol. 2024 Feb 13;58(8):3787–99. doi: 10.1021/acs.est.3c07098 (PMC10902837; doi:10.1021/acs.est.3c07098)
Supplement: Supplementary file 1 — es3c07098_si_001.pdf [file es3c07098_si_001.pdf]

Supporting Information for

Cleaning Up While Changing Gears: The Role of Battery Design, Fossil Fuel Power Plants and Vehicle Policy for Reducing Emissions in the Transition to Electric Vehicles

Matthew B. Bruchon, Zihao Lance Chen, and Jeremy J. Michalek

34 pages, 16 figures, 12 tables*

E-mail: jmichalek@andrew.cmu.edu

Detailed results

Minimum-cost controlled charging

Prior work demonstrates that when charging is optimally controlled to minimize operating costs, the effect on emissions varies depending on scenario.[1] In our 2019 scenario, this style of controlled charging reduces when charging is scheduled to minimize operations costs our BEV externality estimate falls from $8,300 to $7,800 and our PHEV estimate falls from $7,500 to $7,000 (main body Figure 3). This reduction is sufficient to make PHEV consequential emissions favorable relative to ICEVs but not relative to HEVs. Figure 1 shows that for BEVs, controlled charging enables the grid operator to replace some relatively inefficient simple-cycle or combustion engine natural gas generation with more efficient combined cycle natural gas generation. There is also a slight increase in coal generation, which typically results in higher externalities than natural gas, but the slight increase in externalities from fuel switching does not offset the decrease from using more efficient natural gas units. In contrast, in our 2035 scenarios, controlled charging to reduce generation costs can substantially increase or reduce externality costs, depending on the generator fleet, consistent with prior findings in the literature (main body Figures 1 and 4).

Reserve requirements

Our model uses a simplified "N-1" reserve requirement. We test sensitivity to this assumption by running a test case with no reserve requirements and find generator dispatch is not substantially impacted (Figure 16).

Social cost of carbon

As discussed in the main body, SI Figure 2 shows a tradeoff wherein BEVs tend to have higher CAP externalities but lower or similar GHGs relative to other powertrains (across modeling scenario years). This implies that a higher valuation of GHG externalities relative

Figure 1: Sources of new electricity generation induced when 10% of the light-duty passenger car fleet in PJM's service area is replaced with a new BEV with a range of 300 miles. ("Other", including generation such as nuclear, hydroelectric, landfill gas, and petroleum coke, increased by a negligible amount.)

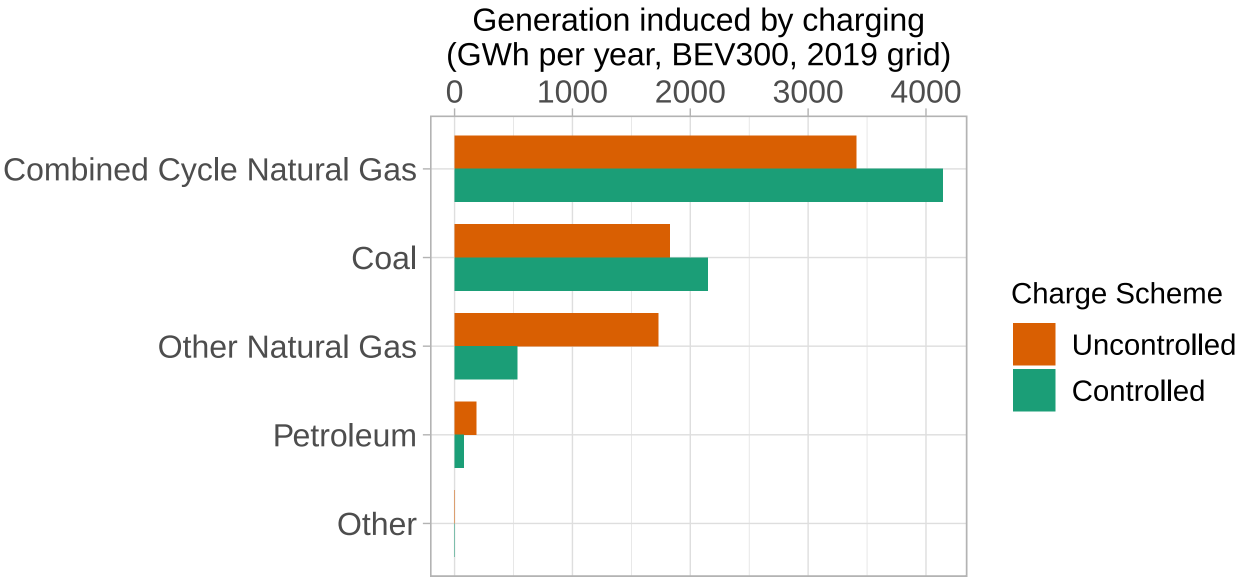

to CAP externalities could change which powertrain's overall externalities are lowest. Recent literature has estimated a higher social cost of carbon of $185 per tonne of CO2 (compared to $51 per tonne used in our base case).[2] Recognizing there is little consensus on what value should be assigned to the social cost of carbon—and, further, acknowledging fundamental challenges in applying traditional social discounting to issues of global change[3,4]—we apply that value as a sensitivity case in order to test a wide range of values. SI Figure 13 shows that when the social cost of carbon is increased to $185, valuation of GHGs externalities are typically larger than CAPs. With these shifts, as shown in SI Figure 14, externality cost estimates increase for all powertrains, and total ICEV externalities rise above BEV externalities for all cases 2019 and beyond, with BEVs producing the lowest total externalities with base case assumptions. SI Figure 15 shows that PHEVs have externalities between ICEVs and HEVs in the 2025 and 2035 base cases.

It is worth noting that this higher social cost of carbon not only changes the rankings of powertrain externalities but also substantially raises externalities across the board. ICEVs,

for example, rise from just over $7,000—already a meaningful value relative to the retail price of a car—to just over $14,000 per car. As the scale of unpriced external costs increases, the justification for policies to address them may also increase.

Policy interactions

Potential policy interactions exist between PEV adoption and federal regulations of light duty fleet fuel economy and greenhouse gas emission rates. Prior work finds that because PEV sales relax an automaker's fleetwide emissions targets, PEV adoption increases overall permitted fleetwide emissions.[5,6] We conduct a post-hoc computation to consider these interactions using the formulation detailed in Jenn et al. (2016), wherein the fleet's compliance-relevant emissions absorb any slack in the standards and rise to the level of maximum permitted fleet emissions.[5] Given that assumption, the effect of replacing an HEV with a PEV is to add the PEV's use phase emissions to the fleet without subtracting the HEV's use phase emissions (because they can "leak" to the rest of the automaker's fleet). This leakage is amplified when sales multiplier incentives are used that treat each PEV sale as more than one sale in compliance calculations, as exist in today's standards. These multiplier incentives change over time and are subject to change in future standards, and we ignore them here. Any sales multiplier incentives would amplify the leakage effects we estimate here.

Adjustments of an automaker's fleet to absorb flexibility of GHG emissions standards may also affect the fleet's criteria emissions as well, but we ignore such possibilities here because vehicles must separately comply with criteria air pollutant regulations.

SI Figure 12 shows results considering those potential policy interactions, which adds around $4100 to each PEV's estimated externalities (for simplicity, we assume here that all PHEV miles are electric, which is a pessimistic assumption for potential policy leakage effects). This increase leads to PEV scenarios that produce larger externalities than those with HEVs or ICEVs across all 2025 and 2035 scenarios. Such policy interactions may change in the future if federal rules change or if fleet emissions drop below the levels mandated by

fleet standards driven by other forces, such that automakers no longer absorb any slack in permitted emissions.

Detailed methods

Future renewable scenarios

For each future scenario, we assign new renewable installations to specific transmission constraint regions within PJM to match the proportions of projects that are currently in PJM's interconnection queue. For the 10% and 22% scenarios, the ratio of installed nameplate capacity of wind versus solar is taken from the same PJM study. Within each technology type, we assume new installations will have capacity factors and generation profiles similar to the historical wind and solar generated in PJM. This assumption is likely optimistic (since more optimal solar and wind sites are more likely to have been built already, all else being equal) and errs on the side of overestimating the load that renewables may offset.

Future generator fleet scenarios

In addition to coal retirements scheduled in EIA-860 forms, we consider cases in which 25% or 50% of remaining installed coal-burning capacity is retired, ordering retirements by oldest plant first (defined as the year the plant came online per NEEDS). For each level of coal retirement, we also consider two cases for natural gas installation: one in which only installs scheduled in EIA-860 forms are included, and one in which retired coal capacity is fully replaced by a random sample of additional new combined-cycle natural gas plants from the PJM interconnection queue. These new natural gas plants are given the heat rate found on average in NEEDS for new combined cycle plant installed since January 2020. The combination of these two changes yields four additional scenarios per year.

Valuation of emissions

Valuation of externalities for CAPs using AP3 requires assigning each unit of emissions to a specific county in the U.S. We locate vehicle tailpipe emissions within the PJM region, proportional to population, and we locate each power plant's combustion emissions at the location of the plant (using stack-level damage factor estimates). For upstream emissions related to extraction and refining of electricity fuels (coal, natural gas, and oil) and vehicular fuels (gasoline), we assume emissions are released at locations based on output-weighted averages of U.S.-based coal mines, natural gas processing plants, and petroleum refineries, respectively.[7–9]

Because this externality valuation method is limited in scope to counties in the U.S., and due to considerable variability and uncertainty in present-day and future locations of each supply chain stage, emissions from automobile and battery manufacture, disposal, and recycling are assumed to originate in U.S. counties with employment in relevant sectors. Locations are further refined using locations of mining facilities for emissions from certain high-emissions materials extraction and refining steps. This method was developed for Bruchon et al. (2021) and is described in greater detail there.[10]

As a simplifying assumption, we treat vehicle disposal and recycling emissions as occurring at the time of vehicle purchase rather than attempting to separate them from manufacturing; these emissions are relatively small.

Modeling assumptions

Some additional modeling decisions and assumptions warrant consideration.

The plant-level emissions factors we use are derived from NEI data for total annual emissions divided by total annual load generation output. This may tend to artificially inflate emissions factors for plants that did more ramping or load-following in 2019 relative to baseload generating units (even if those load-following units serve as baseload in one or

more of our modeling scenarios).

Our future scenarios assume that no changes to transmission constraints or imports or exports will occur relative to our year 2019 data. This may be restrictive, since buildout of additional renewables or other generating capacity may be coupled with additional transmission buildout or with new power purchase agreements between PJM and other balancing regions such as MISO or NYISO.

We assume no change in electrical load vs. year 2019 (except for that induced by the 10% switch to PEVs). A host of factors (such as population growth, regional migration, new mobility services, and shifting weather patterns) all will change electrical demand, both in terms of aggregate levels and within-day demand curves, in ways that may alter optimal generator dispatch.

We model an overnight 10% transition to PEVs, but we do not consider impacts on the used vehicle market or on vehicle scrappage at end of life.[11] Additional investigation is needed to determine how this factor may affect our results.

We do not implement a valuation for CO externalities, for reasons discussed in the Supporting Information of Weis et al. (2016).[12] Excluding a dollar value for CO tends to bias results in favor of gasoline combustion relative to PEVs.

We do not model the emissions-reducing regulations of the EPA's Clean Air Markets Programs, such as the Acid Rain Program, which caps annual NO_X from electricity generating units. At present day, NO_X emissions are low enough that the annual cap is far from binding, and the clearing price of emissions allowances is thus near zero. However, it is conceivable that if the generator fleet changed drastically or other pollutant restrictions were adjusted in the future, such that the electricity sector had additional incentives to avoid new emissions, then the generation induced by new PEV charging may lead to lower consequential emissions.

Figure 2: Evolution over time of estimated consequential life cycle emissions externalities ($ per vehicle lifetime) from criteria pollutants (CAPs) and greenhouse gases (GHGs) for several powertrains for the case with 10% of the light-duty passenger car fleet in PJM's service area replaced with new cars of several powertrain types. "ICEV" denotes a conventional internal combustion engine vehicle, "HEV" denotes a standard gasoline hybrid electric vehicle, "PHEV20" denotes a plug-in hybrid electric vehicle with a battery range of 20 miles, and "BEV300" denotes a battery electric with a battery range of 300 miles. For BEV300 and PHEV20, vertical lines indicate base case estimates (uncontrolled charging, NMC battery chemistry, 10% renewables in 2035, no accelerated coal retirements or natural gas installations). Vertical lines indicate the range of estimates across charging schemes, battery chemistries, and generator fleet makeup (2010 range truncated for readability).

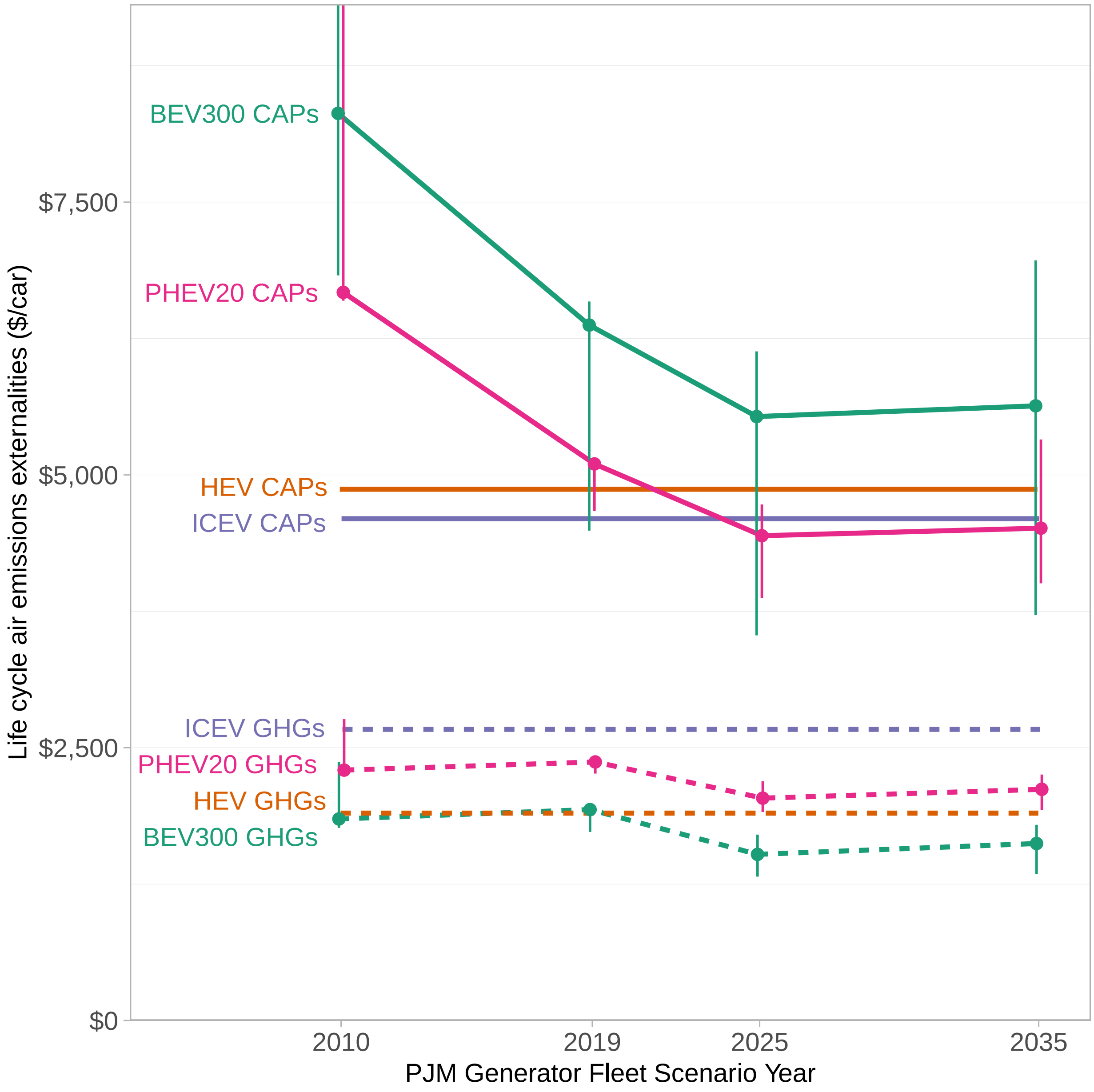

Figure 3: Comparison of consequential emissions externalities ($ per vehicle lifetime) across powertrains for five types of pollutants, assuming 10% of the light-duty passenger car fleet in PJM's service area is replaced with new cars. "ICEV" denotes a conventional (internal combustion) vehicle, "HEV" denotes a standard hybrid electric vehicle, "PHEV" denotes a plug-in hybrid electric vehicle with a battery range of 20 miles, and "BEV" denotes a pure battery electric with a battery range of 300 miles. "CC" indicates that battery charging is optimally scheduled by PJM to minimize system operations costs, and "UC" indicates that battery charging is uncontrolled (i.e., initiated by the vehicle owner as soon as they complete their daily driving and arrive home).

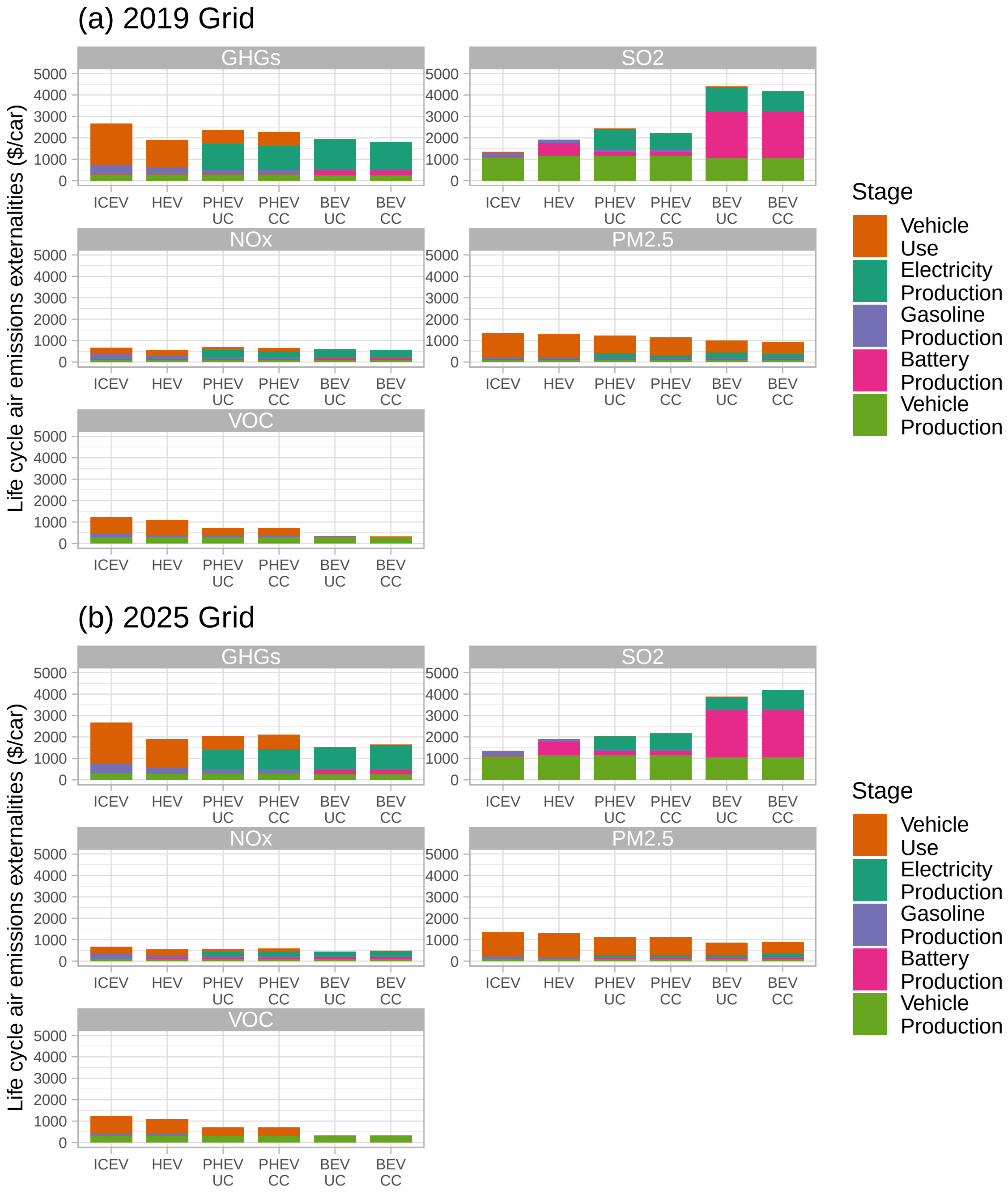

Figure 4: Comparison of consequential emissions externalities ($ per vehicle lifetime) across powertrains for five types of pollutants, assuming 10% of the light-duty passenger car fleet in PJM's service area is replaced with new cars. "ICEV" denotes a conventional (internal combustion) vehicle, "HEV" denotes a standard hybrid electric vehicle, "PHEV" denotes a plug-in hybrid electric vehicle with a battery range of 20 miles, and "BEV" denotes a pure battery electric with a battery range of 300 miles. "CC" indicates that battery charging is optimally scheduled by PJM to minimize system operations costs, and "UC" indicates that battery charging is uncontrolled (i.e., initiated by the vehicle owner as soon as they complete their daily driving and arrive home).

(c) Future Grid (10% Wind+Solar)

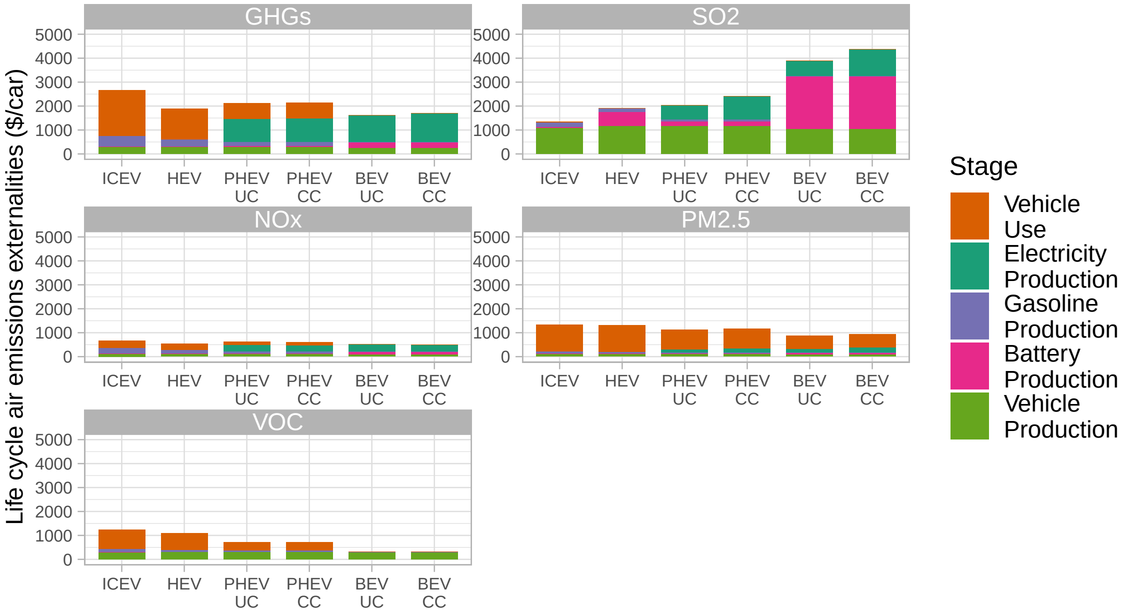

(d) Future Grid (22% Wind+Solar)

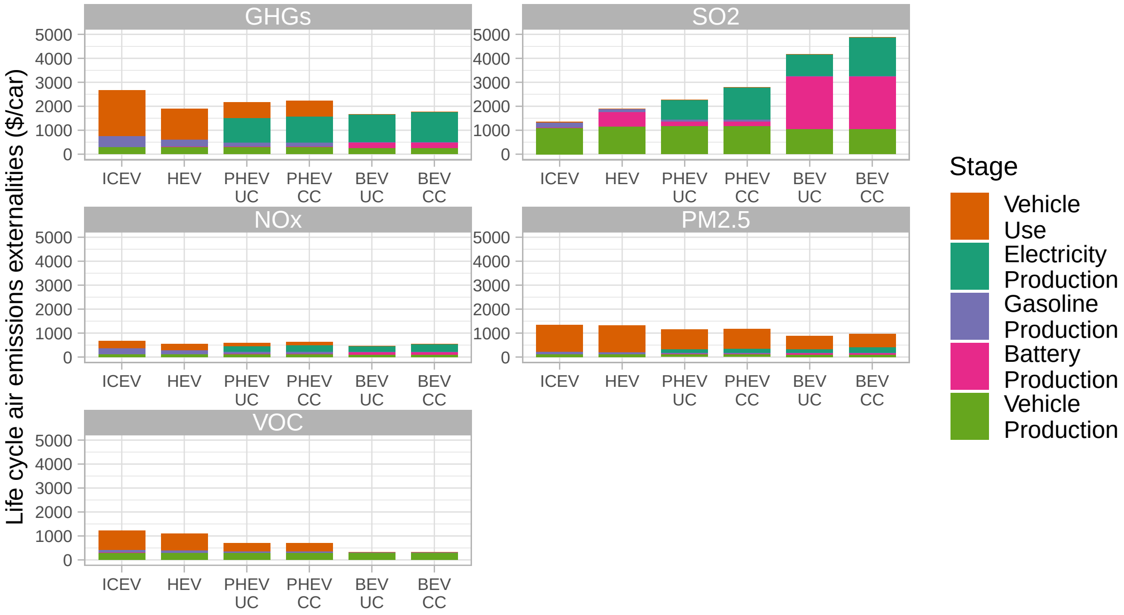

Figure 5: Comparison of consequential emissions (lb per vehicle lifetime) across powertrains for six types of pollutants, assuming 10% of the light-duty passenger car fleet in PJM's service area is replaced with new cars. "ICEV" denotes a conventional (internal combustion) vehicle, "HEV" denotes a standard hybrid electric vehicle, "PHEV" denotes a plug-in hybrid electric vehicle with a battery range of 20 miles, and "BEV" denotes a pure battery electric with a battery range of 300 miles. "CC" indicates that battery charging is optimally scheduled by PJM to minimize system operations costs, and "UC" indicates that battery charging is uncontrolled (i.e., initiated by the vehicle owner as soon as they complete their daily driving and arrive home).

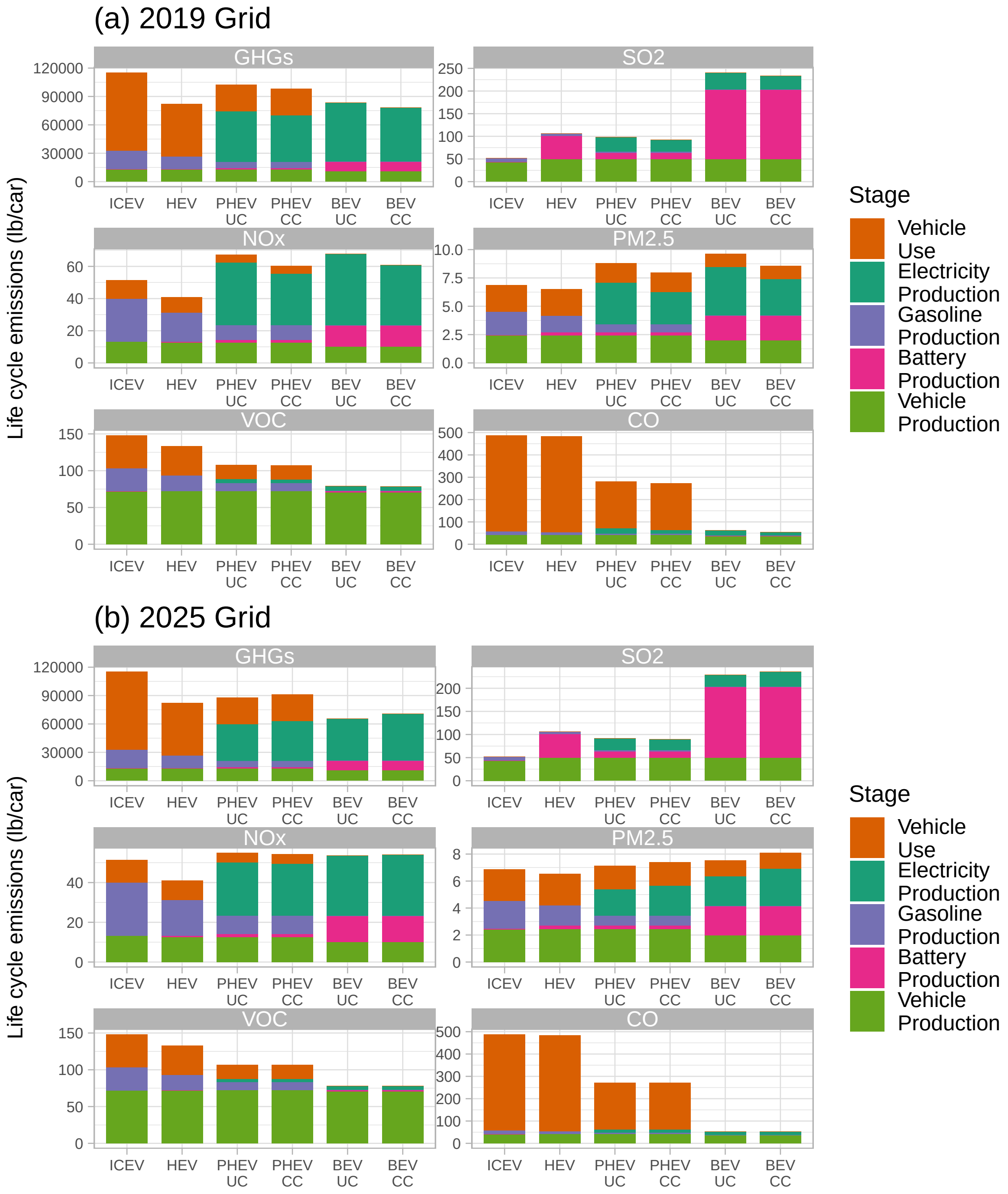

Figure 6: Comparison of consequential emissions (lb per vehicle lifetime) across powertrains for six types of pollutants, assuming 10% of the light-duty passenger car fleet in PJM's service area is replaced with new cars. "ICEV" denotes a conventional (internal combustion) vehicle, "HEV" denotes a standard hybrid electric vehicle, "PHEV" denotes a plug-in hybrid electric vehicle with a battery range of 20 miles, and "BEV" denotes a pure battery electric with a battery range of 300 miles. "CC" indicates that battery charging is optimally scheduled by PJM to minimize system operations costs, and "UC" indicates that battery charging is uncontrolled (i.e., initiated by the vehicle owner as soon as they complete their daily driving and arrive home).

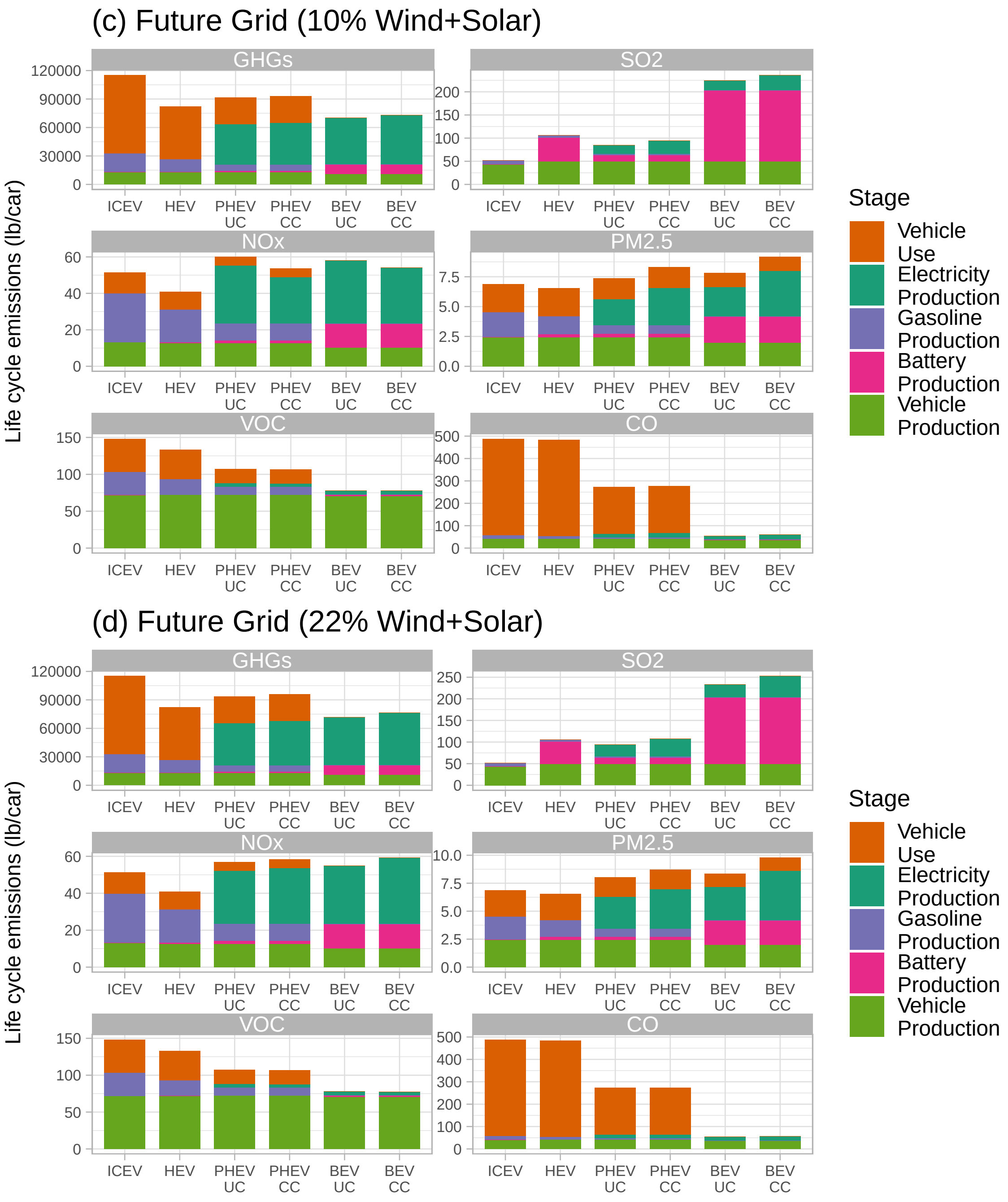

Figure 7: Life cycle emissions externalities per vehicle in 2019 if BEVs and PHEVs use the LFP battery chemistry, assuming 10% of the light-duty passenger car fleet in PJM's service area is replaced with new cars. "ICEV" denotes a conventional (internal combustion) vehicle, “HEV” denotes a standard hybrid electric vehicle, “PHEV20” denotes a plug-in hybrid electric vehicle with a battery range of 20 miles, and “BEV300” denotes a pure battery electric with a battery range of 300 miles. “CC” indicates that battery charging is optimally scheduled by PJM to minimize system operations costs, and “UC” indicates that battery charging is uncontrolled (i.e., initiated by the vehicle owner as soon as they complete their daily driving and arrive home “ Manufacture” also includes disposal and recycling; “Vehicle Use” includes tailpipe emissions and tire and brake wear.).

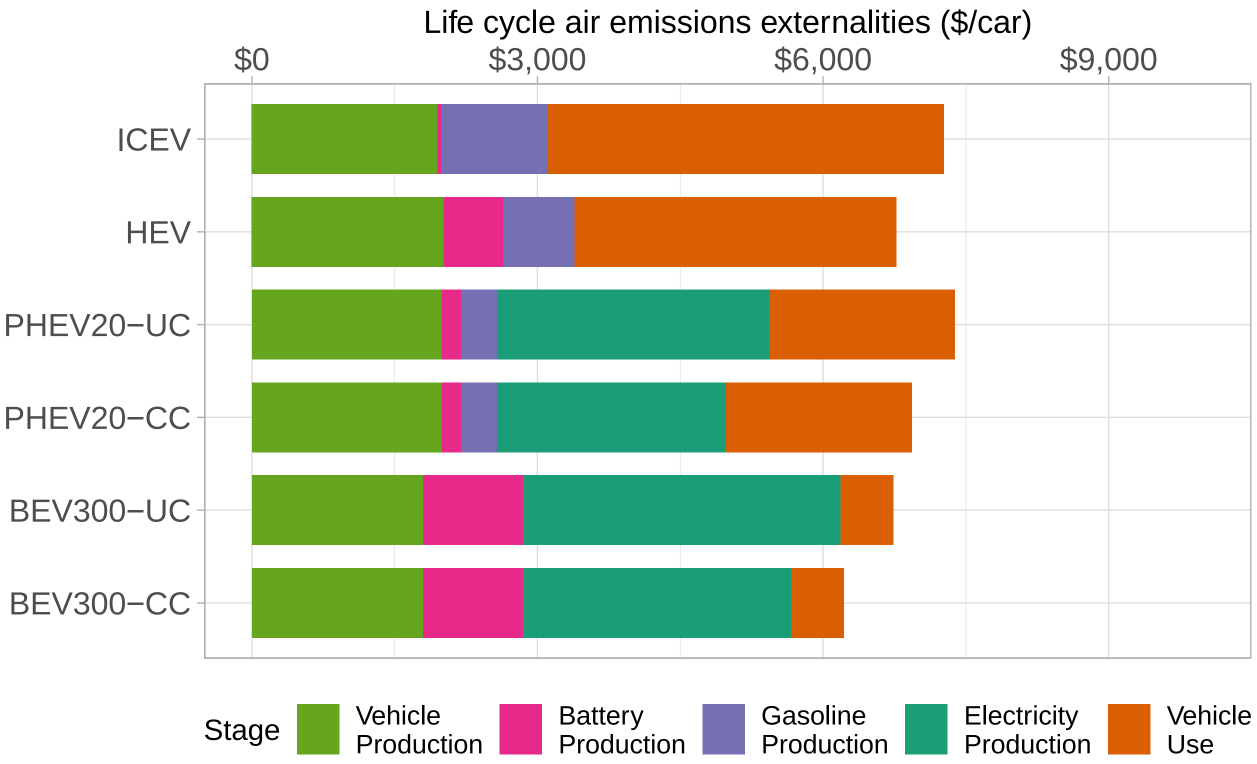

Figure 8: Life cycle emissions externalities per vehicle in 2025, assuming 10% of the light-duty passenger car fleet in PJM's service area is replaced with new cars. "ICEV" denotes a conventional (internal combustion) vehicle, "HEV" denotes a standard hybrid electric vehicle, "PHEV20" denotes a plug-in hybrid electric vehicle with a battery range of 20 miles, and "BEV300" denotes a pure battery electric with a battery range of 300 miles. "CC" indicates that battery charging is optimally scheduled by PJM to minimize system operations costs, and "UC" indicates that battery charging is uncontrolled (i.e., initiated by the vehicle owner as soon as they complete their daily driving and arrive home " Manufacture" also includes disposal and recycling; "Vehicle Use" includes tailpipe emissions and tire and brake wear.).

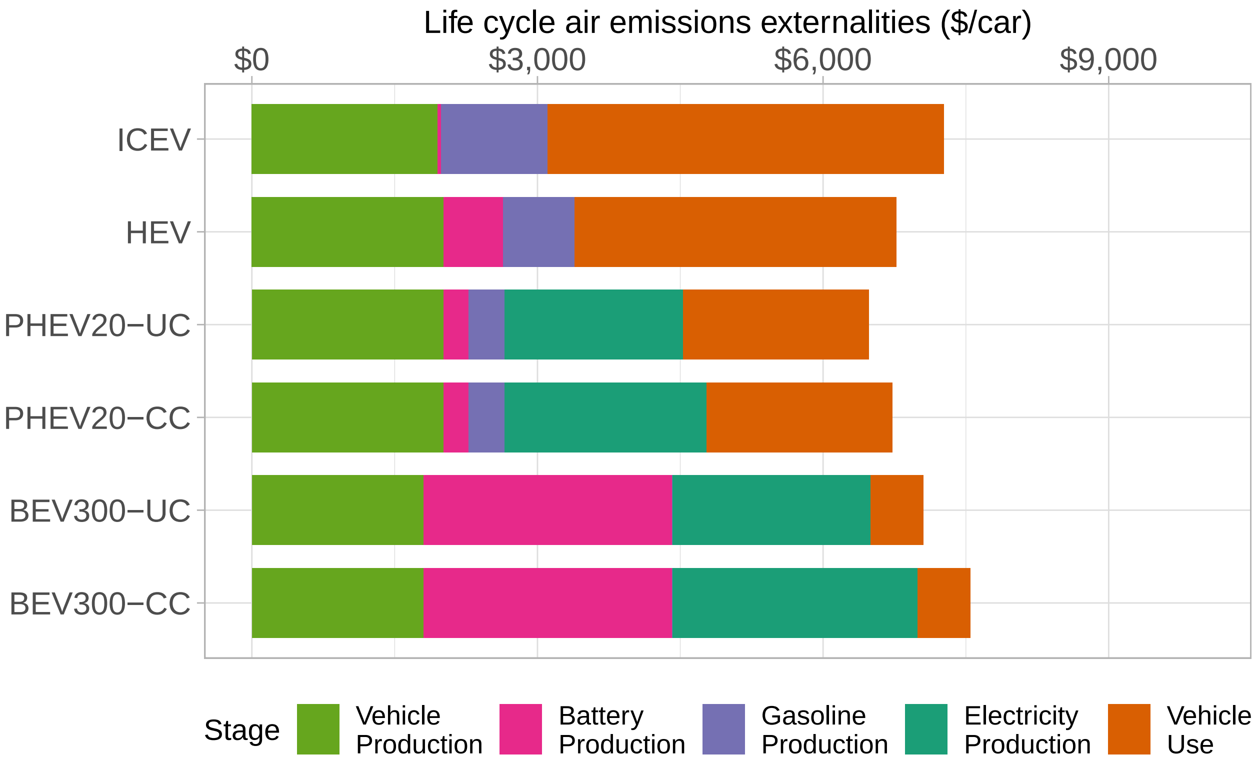

Figure 9: Life cycle emissions externalities ($ per vehicle lifetime) in our 2035 base case (10% renewable energy), assuming 10% of the light-duty passenger car fleet in PJM's service area is replaced with new cars. "ICEV" denotes a conventional (internal combustion) vehicle, "HEV" denotes a standard hybrid electric vehicle, "PHEV 20" denotes a plug-in hybrid electric vehicle with a battery range of 20 miles, and "BEV 300" denotes a pure battery electric with a battery range of 300 miles. "CC" indicates that battery charging is optimally scheduled by PJM to minimize system operations costs, and "UC" indicates that battery charging is uncontrolled (i.e., initiated by the vehicle owner as soon as they complete their daily driving and arrive home). "Manufacture" also includes disposal and recycling; "Vehicle Use" includes tailpipe emissions and tire and brake wear.).

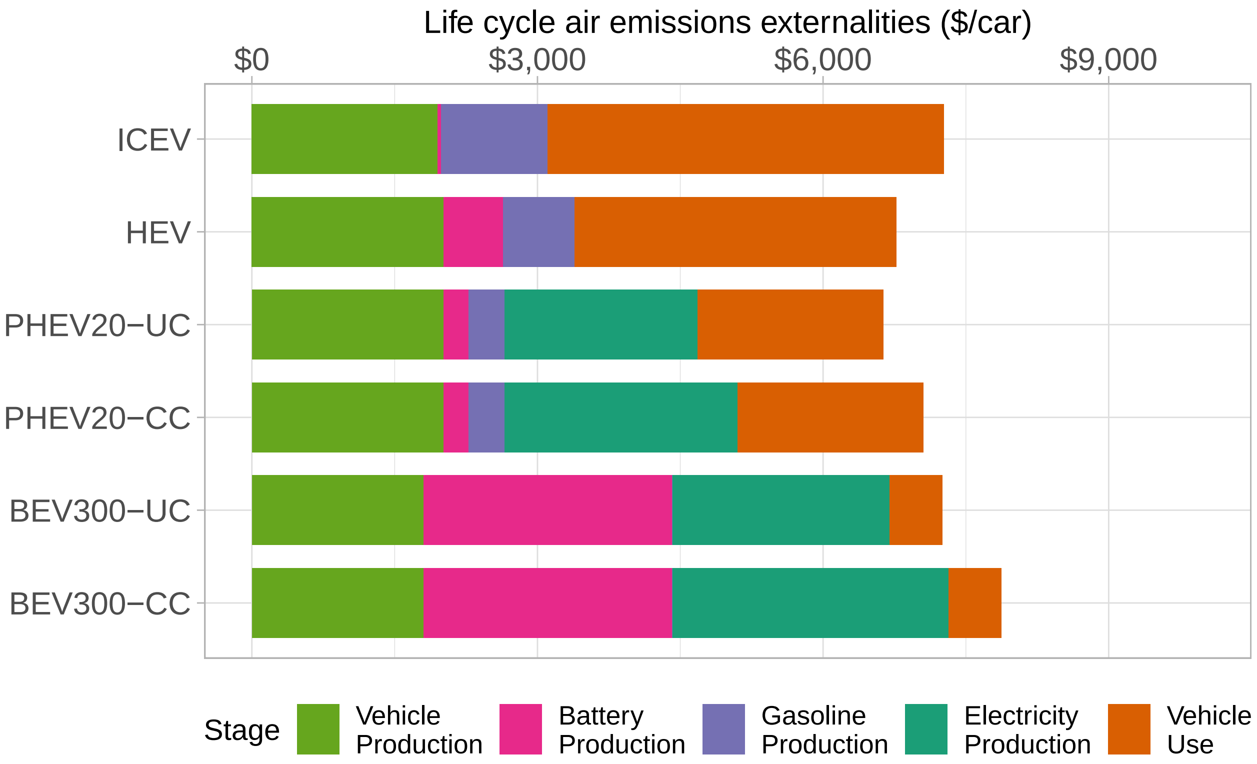

Figure 10: Life cycle emissions externalities ($ per vehicle lifetime) in our future 22% renewable energy scenario, assuming 10% of the light-duty passenger car fleet in PJM's service area is replaced with new cars. "ICEV" denotes a conventional (internal combustion) vehicle, "HEV" denotes a standard hybrid electric vehicle, "PHEV 20" denotes a plug-in hybrid electric vehicle with a battery range of 20 miles, and "BEV 300" denotes a pure battery electric with a battery range of 300 miles. "CC" indicates that battery charging is optimally scheduled by PJM to minimize system operations costs, and "UC" indicates that battery charging is uncontrolled (i.e., initiated by the vehicle owner as soon as they complete their daily driving and arrive home). "Manufacture" also includes disposal and recycling; "Vehicle Use" includes tailpipe emissions and tire and brake wear.).

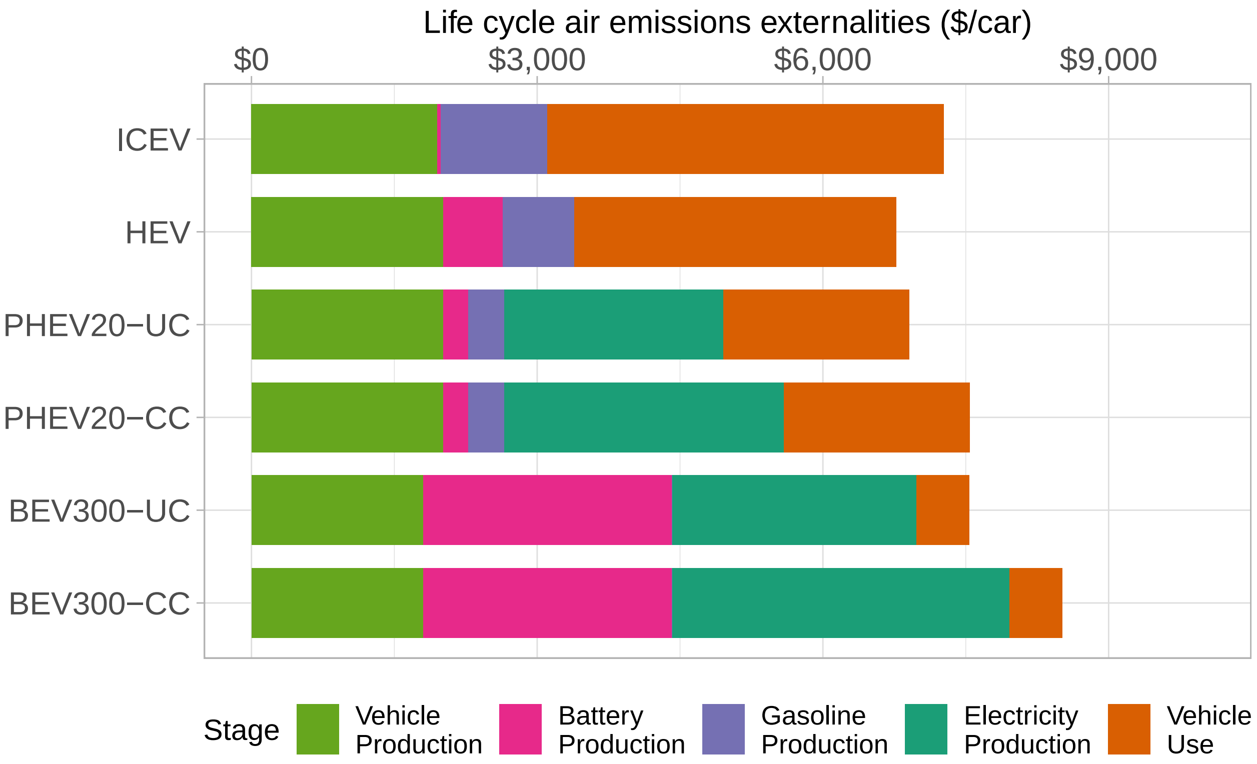

Figure 11: Sources of new electricity generation induced in our 2035 base case (10% renewable energy) when 10% of the light-duty passenger car fleet in PJM's service area is replaced with a new BEV with a range of 300 miles. ("Other", including generation such as nuclear, hydroelectric, landfill gas, and petroleum coke, increased by a negligible amount.

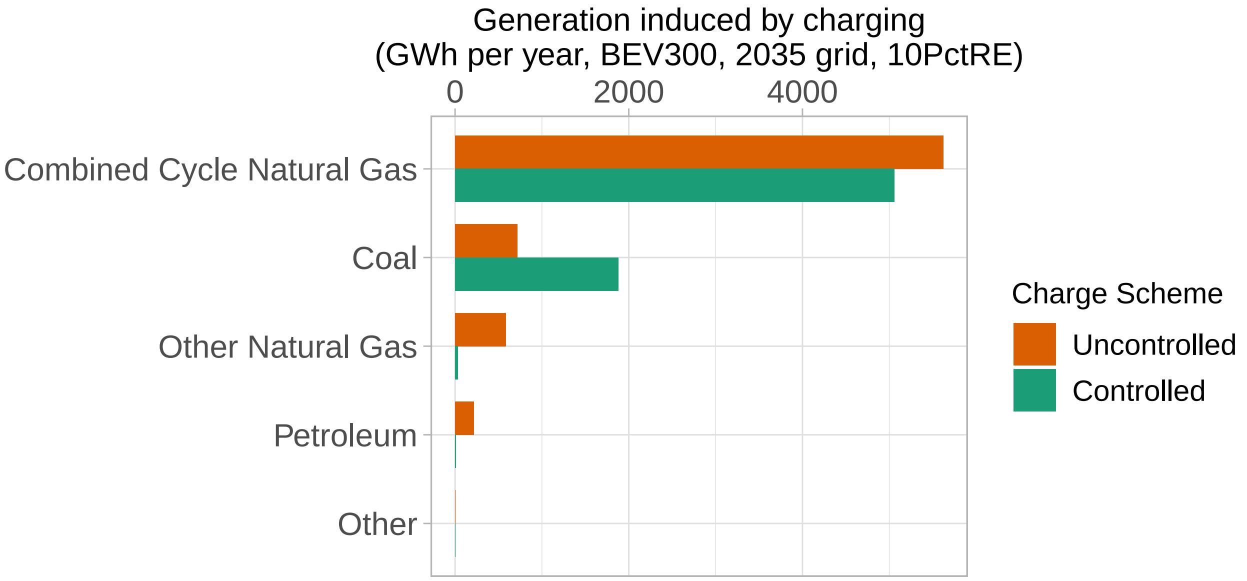

References

(1) Weis, A.; Michalek, J. J.; Jaramillo, P.; Lueken, R. Emissions and cost implications of controlled electric vehicle charging in the U.S. PJM interconnection. *Environmental Science & Technology* **2015**, *49*, 5813–5819.

(2) Rennert, K. et al. Comprehensive Evidence Implies a Higher Social Cost of CO2. *Nature 2022* **2022**, *1*, 1–3.

(3) Morgan, M. G.; Kandlikar, M.; Risbey, J.; Dowlatabadi, H. Why Conventional Tools for Policy Analysis Are Often Inadequate for Problems of Global Change. *Climatic Change* **1999**, *41*, 271–281.

(4) Kaufman, N. *How the unequal effects of climate change broke economists' favorite tool*; 2022; pp 1–12.

(5) Jenn, A.; Azevedo, I. M. L.; Michalek, J. J. Alternative Fuel Vehicle Adoption Increases Fleet Gasoline Consumption and Greenhouse Gas Emissions under United States Cor-

Figure 12: Range of life cycle emissions externalities ($ per vehicle lifetime) relative to an HEV for the base case power grid for each modeling year, with and without consideration of potential policy interactions from federal regulations of corporate average fuel economy and greenhouse gas emission standards. "ICEV" denotes a conventional internal combustion engine vehicle, "PHEV20" denotes a plug-in hybrid electric vehicle with a battery range of 20 miles, and "BEV300" denotes a battery electric with a battery range of 300 miles; "UC" and "CC" indicate uncontrolled and controlled charging schemes, respectively. Error bars are the range of values across battery chemistries. For both PHEVs and BEVs, LFP is the lowest-externality chemistry. For PHEVs, NMC111 is the highest (and the base case). For BEVs, NMC622 is the base case and NMC811 is the highest.

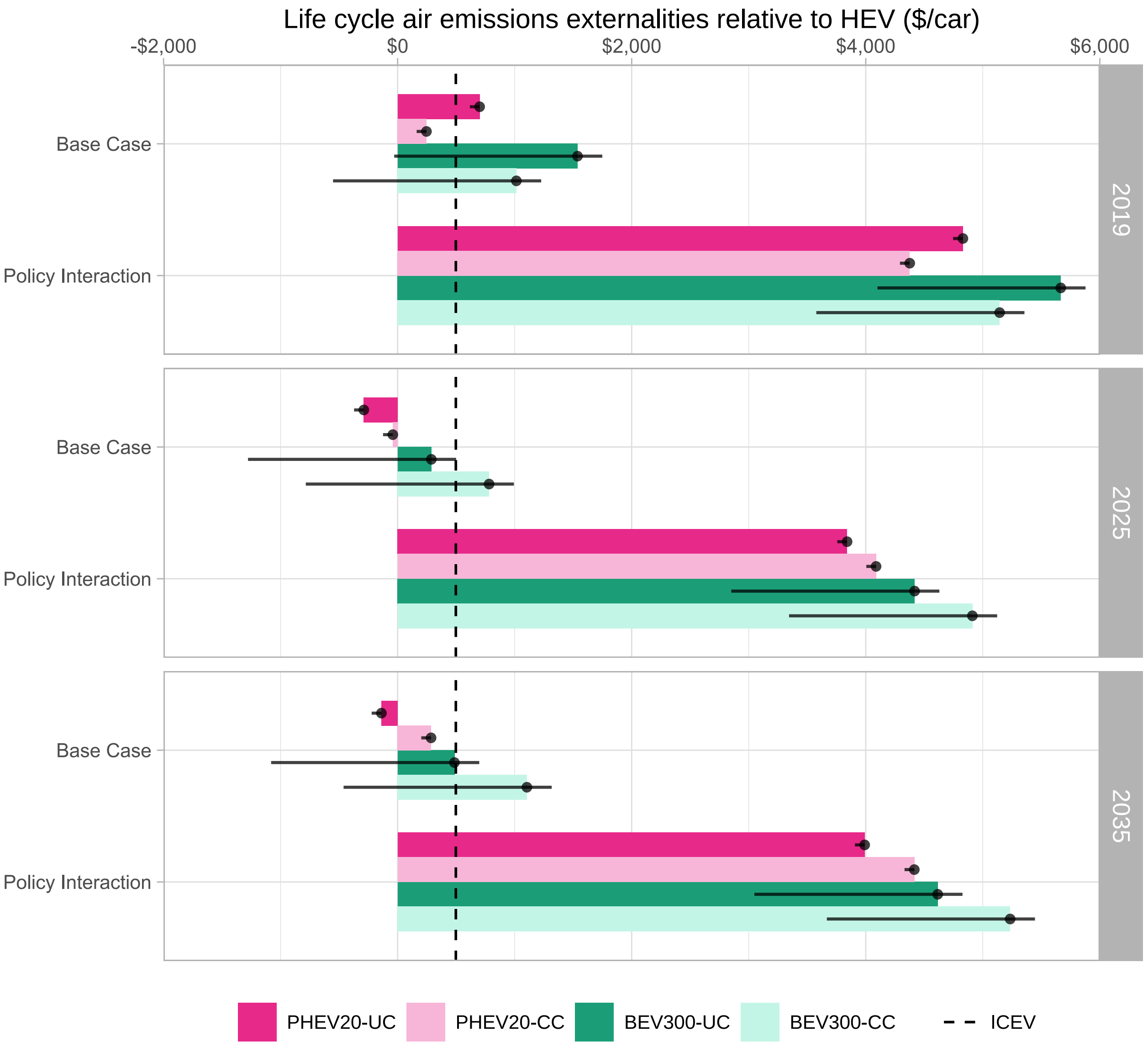

Figure 13: Evolution over time, using a alternative social cost of carbon value of \$185,[2] of estimated consequential life cycle emissions externalities from criteria pollutants (CAPs, solid lines) and greenhouse gases (GHGs, dashed lines) for several powertrains for the case with 10% of the light-duty passenger car fleet in PJM's service area replaced with new cars of several powertrain types. "ICEV" denotes a conventional internal combustion engine vehicle, "HEV" denotes a standard gasoline hybrid electric vehicle, "PHEV20" denotes a plug-in hybrid electric vehicle with a battery range of 20 miles, and "BEV300" denotes a battery electric with a battery range of 300 miles. For BEV300 and PHEV20, vertical lines indicate base case estimates (uncontrolled charging, NMC battery chemistry, 10% renewables in 2035, no accelerated coal retirements or natural gas installations). Vertical lines indicate the range of estimates across charging schemes, battery chemistries, and generator fleet makeup (2010 range truncated for readability).

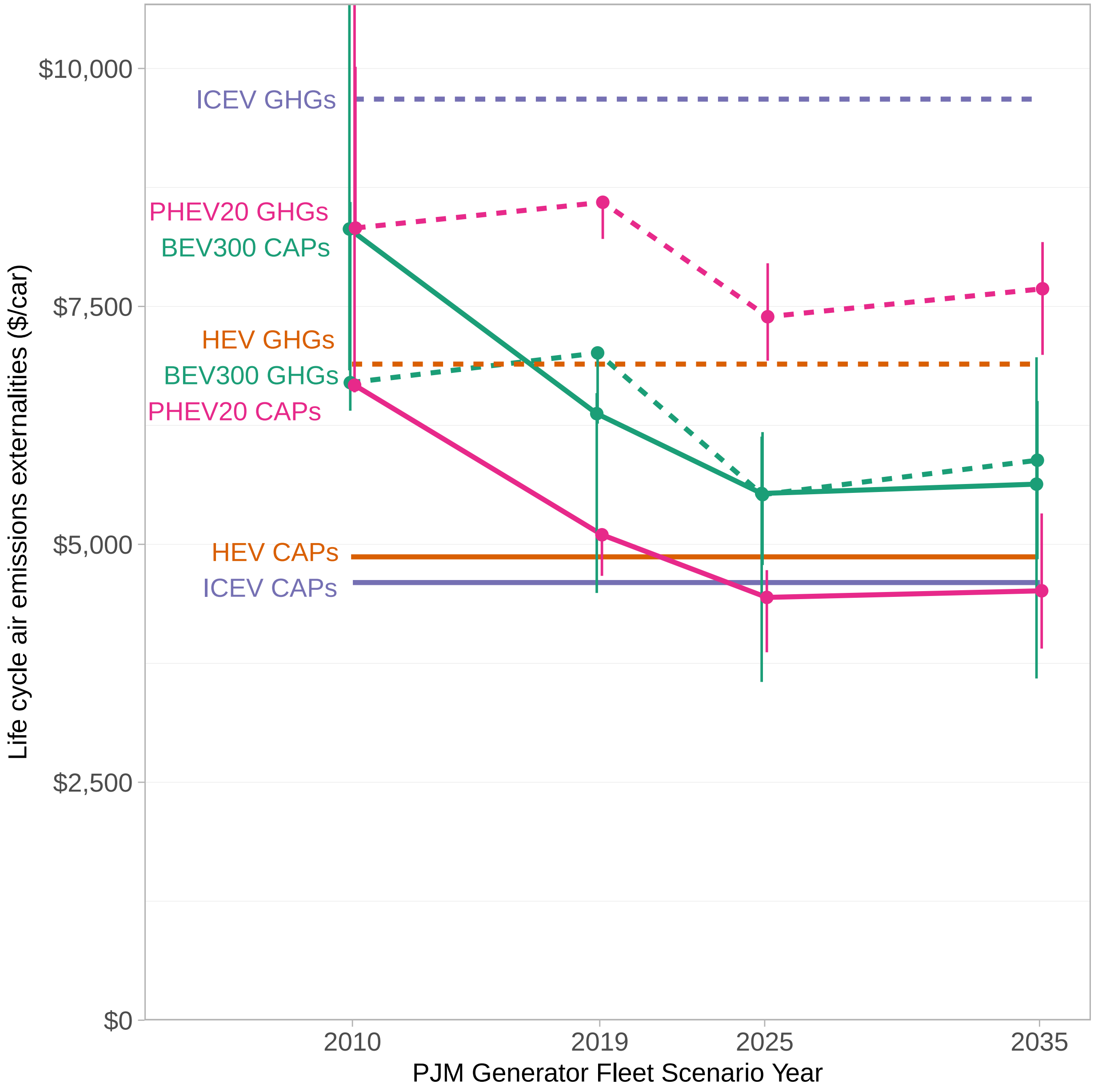

Figure 14: Change in estimated consequential life cycle emissions externalities ($ per vehicle lifetime), using a alternative social cost of carbon value of $185,[2] as PJM's generator fleet evolves over time for several powertrains for the case where 10% of the light-duty passenger car fleet in PJM's service area is replaced with new cars of several powertrain types: "ICEV" = conventional internal combustion engine vehicle, "HEV" = gasoline hybrid electric vehicle, and "BEV300" = battery electric with a battery range of 300 miles. For BEV300, the base case includes uncontrolled charging, NMC622 battery chemistry, 10% renewables in 2035, and no accelerated coal retirements or natural gas installations. The y-axis is truncated to make trends more visible.

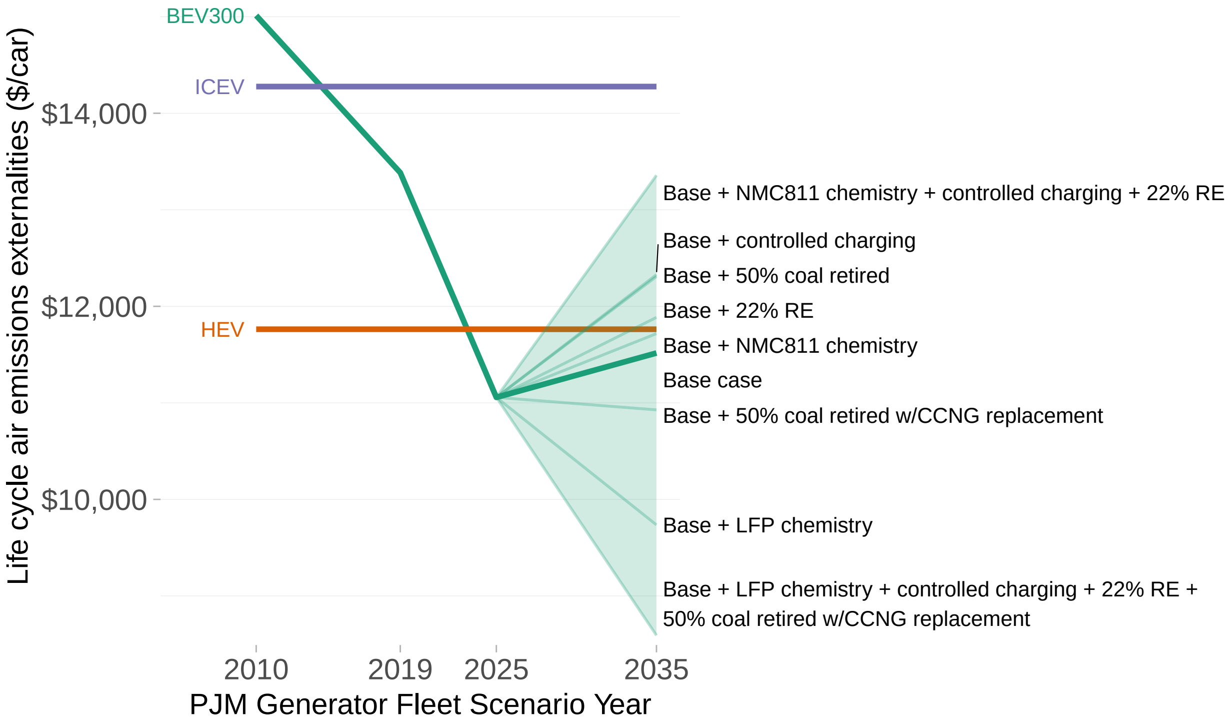

Figure 15: Change in estimated consequential life cycle emissions externalities, using a alternative social cost of carbon value of \$185,[2] as PJM's generator fleet evolves over time for several powertrains for the case where 10% of the light-duty passenger car fleet in PJM's service area is replaced with new cars of several powertrain types: "ICEV" = conventional internal combustion engine vehicle, "HEV" = gasoline hybrid electric vehicle, "PHEV20" = plug-in hybrid electric vehicle with a battery range of 20 miles. For PHEV20, the base case includes uncontrolled charging, NMC111 battery chemistry, 10% renewables in 2035, and no accelerated coal retirements or natural gas installations.

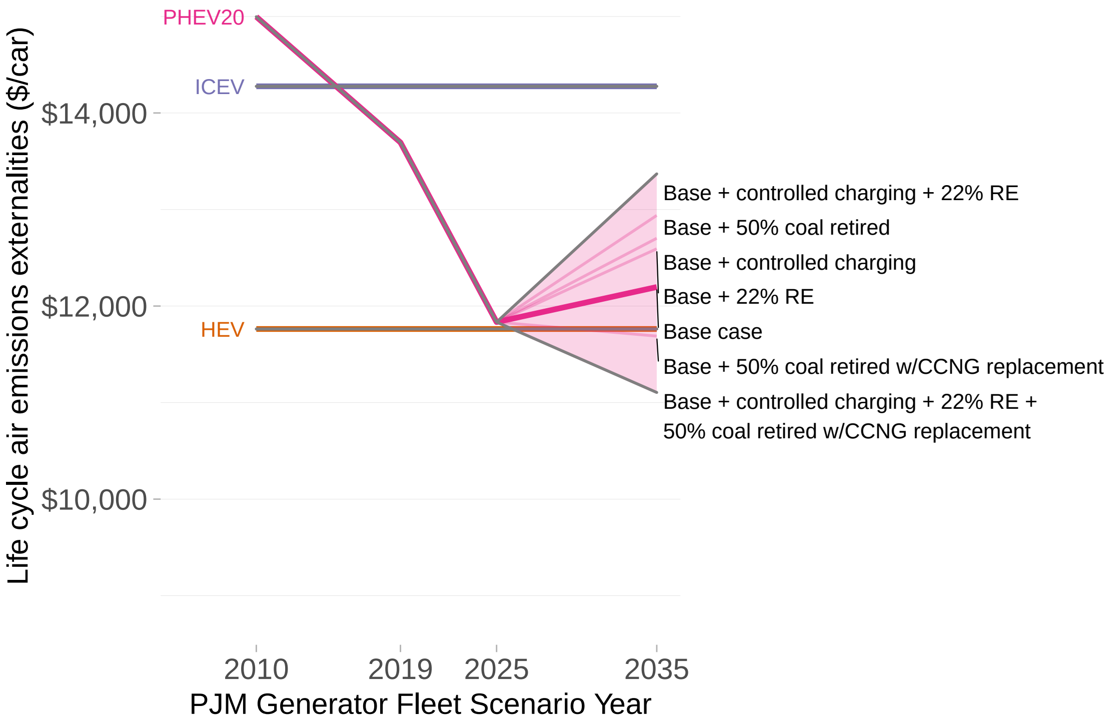

Table 1: Consequential emissions externalities in 2019 ($ per vehicle lifetime) across powertrains for five types of pollutants, assuming 10% of the light-duty passenger car fleet in PJM's service area is replaced with new cars. "ICEV" denotes a conventional (internal combustion) vehicle, "HEV" denotes a standard hybrid electric vehicle, "PHEV20" denotes a plug-in hybrid electric vehicle with a battery range of 20 miles, and "BEV300" denotes a pure battery electric with a battery range of 300 miles. "CC" indicates that battery charging is optimally scheduled by PJM to minimize system operations costs, and "UC" indicates that battery charging is uncontrolled (i.e., initiated by the vehicle owner as soon as they complete their daily driving and arrive home).

			**PHEV20**		**BEV300**	
	**ICEV**	**HEV**	**UC**	**CC**	**UC**	**CC**
**Total**	**7,266**	**6,769**	**7,444**	**7,001**	**8,277**	**7,767**
**GHGs**	**2,668**	**1,900**	**2,369**	**2,270**	**1,933**	**1,809**
Vehicle Production	305	297	298	298	250	250
Battery Production	2	16	34	34	239	239
Electricity Production	-	-	1,235	1,136	1,444	1,320
Gasoline Production	448	301	152	152	-	-
Vehicle Use	1,913	1,286	650	650	-	-
**SO2**	**1,352**	**1,907**	**2,429**	**2,223**	**4,398**	**4,157**
Vehicle Production	1,087	1,161	1,165	1,165	1,050	1,050
Battery Production	31	598	205	205	2,191	2,191
Electricity Production	-	-	989	783	1,157	916
Gasoline Production	204	134	64	64	-	-
Vehicle Use	30	15	7	7	-	-
**PM2.5**	**1,337**	**1,314**	**1,229**	**1,146**	**1,008**	**912**
Vehicle Production	125	125	125	125	102	102
Battery Production	3	8	9	9	71	71
Electricity Production	-	-	240	158	279	182
Gasoline Production	96	69	33	33	-	-
Vehicle Use	1,113	1,113	822	822	557	557
**VOC**	**1,228**	**1,095**	**714**	**710**	**331**	**327**
Vehicle Production	308	308	308	308	301	301
Battery Production	0	1	1	1	6	6
Electricity Production	-	-	20	17	23	19
Gasoline Production	125	84	42	42	-	-
Vehicle Use	795	702	342	342	-	-
**NOx**	**682**	**553**	**703**	**651**	**607**	**562**
Vehicle Production	127	121	121	121	98	98
Battery Production	1	6	14	14	107	107
Electricity Production	-	-	356	304	402	357
Gasoline Production	245	164	84	84	-	-
Vehicle Use	309	262	129	129	-	-

Table 2: Consequential emissions externalities in 2025 ($ per vehicle lifetime) across powertrains for five types of pollutants, assuming 10% of the light-duty passenger car fleet in PJM's service area is replaced with new cars. "ICEV" denotes a conventional (internal combustion) vehicle, "HEV" denotes a standard hybrid electric vehicle, "PHEV20" denotes a plug-in hybrid electric vehicle with a battery range of 20 miles, and "BEV300" denotes a pure battery electric with a battery range of 300 miles. "CC" indicates that battery charging is optimally scheduled by PJM to minimize system operations costs, and "UC" indicates that battery charging is uncontrolled (i.e., initiated by the vehicle owner as soon as they complete their daily driving and arrive home).

			**PHEV20**		**BEV300**	
	**ICEV**	**HEV**	**UC**	**CC**	**UC**	**CC**
**Total**	**7,266**	**6,769**	**6,455**	**6,703**	**7,028**	**7,523**
**GHGs**	**2,668**	**1,900**	**2,038**	**2,110**	**1,522**	**1,635**
Vehicle Production	305	297	298	298	250	250
Battery Production	2	16	34	34	239	239
Electricity Production	-	-	904	977	1,033	1,146
Gasoline Production	448	301	152	152	-	-
Vehicle Use	1,913	1,286	650	650	-	-
**SO2**	**1,352**	**1,907**	**2,039**	**2,177**	**3,874**	**4,193**
Vehicle Production	1,087	1,161	1,165	1,165	1,050	1,050
Battery Production	31	598	205	205	2,191	2,191
Electricity Production	-	-	598	736	633	952
Gasoline Production	204	134	64	64	-	-
Vehicle Use	30	15	7	7	-	-
**PM2.5**	**1,337**	**1,314**	**1,107**	**1,114**	**859**	**880**
Vehicle Production	125	125	125	125	102	102
Battery Production	3	8	9	9	71	71
Electricity Production	-	-	118	126	129	150
Gasoline Production	96	69	33	33	-	-
Vehicle Use	1,113	1,113	822	822	557	557
**VOC**	**1,228**	**1,095**	**710**	**709**	**326**	**326**
Vehicle Production	308	308	308	308	301	301
Battery Production	0	1	1	1	6	6
Electricity Production	-	-	16	16	19	18
Gasoline Production	125	84	42	42	-	-
Vehicle Use	795	702	342	342	-	-
**NOx**	**682**	**553**	**562**	**593**	**446**	**489**
Vehicle Production	127	121	121	121	98	98
Battery Production	1	6	14	14	107	107
Electricity Production	-	-	215	246	241	284
Gasoline Production	245	164	84	84	-	-
Vehicle Use	309	262	129	129	-	-

Table 3: Consequential emissions externalities ($ per vehicle lifetime) in 2035, assuming 10% renewable energy generation, across powertrains for five types of pollutants, assuming 10% of the light-duty passenger car fleet in PJM's service area is replaced with new cars. "ICEV" denotes a conventional (internal combustion) vehicle, "HEV" denotes a standard hybrid electric vehicle, "PHEV20" denotes a plug-in hybrid electric vehicle with a battery range of 20 miles, and "BEV300" denotes a pure battery electric with a battery range of 300 miles. "CC" indicates that battery charging is optimally scheduled by PJM to minimize system operations costs, and "UC" indicates that battery charging is uncontrolled (i.e., initiated by the vehicle owner as soon as they complete their daily driving and arrive home).

			**PHEV20**		**BEV300**	
	**ICEV**	**HEV**	**UC**	**CC**	**UC**	**CC**
**Total**	**7,266**	**6,769**	**6,607**	**7,032**	**7,228**	**7,849**
**GHGs**	**2,668**	**1,900**	**2,119**	**2,150**	**1,622**	**1,695**
Vehicle Production	305	297	298	298	250	250
Battery Production	2	16	34	34	239	239
Electricity Production	-	-	985	1,016	1,133	1,206
Gasoline Production	448	301	152	152	-	-
Vehicle Use	1,913	1,286	650	650	-	-
**SO2**	**1,352**	**1,907**	**2,034**	**2,403**	**3,892**	**4,370**
Vehicle Production	1,087	1,161	1,165	1,165	1,050	1,050
Battery Production	31	598	205	205	2,191	2,191
Electricity Production	-	-	594	963	651	1,129
Gasoline Production	204	134	64	64	-	-
Vehicle Use	30	15	7	7	-	-
**PM2.5**	**1,337**	**1,314**	**1,120**	**1,174**	**879**	**952**
Vehicle Production	125	125	125	125	102	102
Battery Production	3	8	9	9	71	71
Electricity Production	-	-	131	185	149	222
Gasoline Production	96	69	33	33	-	-
Vehicle Use	1,113	1,113	822	822	557	557
**VOC**	**1,228**	**1,095**	**710**	**709**	**327**	**326**
Vehicle Production	308	308	308	308	301	301
Battery Production	0	1	1	1	6	6
Electricity Production	-	-	17	16	19	18
Gasoline Production	125	84	42	42	-	-
Vehicle Use	795	702	342	342	-	-
**NOx**	**682**	**553**	**624**	**597**	**508**	**506**
Vehicle Production	127	121	121	121	98	98
Battery Production	1	6	14	14	107	107
Electricity Production	-	-	277	249	304	301
Gasoline Production	245	164	84	84	-	-
Vehicle Use	309	262	129	129	-	-

Table 4: Consequential emissions externalities ($ per vehicle lifetime) in 2035, assuming 22% renewable energy generation, across powertrains for five types of pollutants, assuming 10% of the light-duty passenger car fleet in PJM's service area is replaced with new cars. "ICEV" denotes a conventional (internal combustion) vehicle, "HEV" denotes a standard hybrid electric vehicle, "PHEV20" denotes a plug-in hybrid electric vehicle with a battery range of 20 miles, and "BEV300" denotes a pure battery electric with a battery range of 300 miles. "CC" indicates that battery charging is optimally scheduled by PJM to minimize system operations costs, and "UC" indicates that battery charging is uncontrolled (i.e., initiated by the vehicle owner as soon as they complete their daily driving and arrive home).

			**PHEV20**		**BEV300**	
	**ICEV**	**HEV**	**UC**	**CC**	**UC**	**CC**
**Total**	**7,266**	**6,769**	**6,883**	**7,529**	**7,511**	**8,501**
**GHGs**	**2,668**	**1,900**	**2,165**	**2,218**	**1,655**	**1,766**
Vehicle Production	305	297	298	298	250	250
Battery Production	2	16	34	34	239	239
Electricity Production	-	-	1,031	1,084	1,166	1,277
Gasoline Production	448	301	152	152	-	-
Vehicle Use	1,913	1,286	650	650	-	-
**SO2**	**1,352**	**1,907**	**2,280**	**2,787**	**4,171**	**4,887**
Vehicle Production	1,087	1,161	1,165	1,165	1,050	1,050
Battery Production	31	598	205	205	2,191	2,191
Electricity Production	-	-	840	1,347	930	1,646
Gasoline Production	204	134	64	64	-	-
Vehicle Use	30	15	7	7	-	-
**PM2.5**	**1,337**	**1,314**	**1,145**	**1,180**	**893**	**972**
Vehicle Production	125	125	125	125	102	102
Battery Production	3	8	9	9	71	71
Electricity Production	-	-	156	192	163	242
Gasoline Production	96	69	33	33	-	-
Vehicle Use	1,113	1,113	822	822	557	557
**VOC**	**1,228**	**1,095**	**710**	**708**	**326**	**325**
Vehicle Production	308	308	308	308	301	301
Battery Production	0	1	1	1	6	6
Electricity Production	-	-	16	15	19	17
Gasoline Production	125	84	42	42	-	-
Vehicle Use	795	702	342	342	-	-
**NOx**	**682**	**553**	**583**	**636**	**465**	**552**
Vehicle Production	127	121	121	121	98	98
Battery Production	1	6	14	14	107	107
Electricity Production	-	-	236	289	261	347
Gasoline Production	245	164	84	84	-	-
Vehicle Use	309	262	129	129	-	-

Table 5: Consequential emissions in 2019 (lb per vehicle lifetime) across powertrains for six types of pollutants, assuming 10% of the light-duty passenger car fleet in PJM's service area is replaced with new cars. "ICEV" denotes a conventional (internal combustion) vehicle, "HEV" denotes a standard hybrid electric vehicle, "PHEV20" denotes a plug-in hybrid electric vehicle with a battery range of 20 miles, and "BEV300" denotes a pure battery electric with a battery range of 300 miles. "CC" indicates that battery charging is optimally scheduled by PJM to minimize system operations costs, and "UC" indicates that battery charging is uncontrolled (i.e., initiated by the vehicle owner as soon as they complete their daily driving and arrive home).

			**PHEV20**		**BEV300**	
	**ICEV**	**HEV**	**UC**	**CC**	**UC**	**CC**
**GHGs**	**115,330.3**	**82,152.9**	**102,416.0**	**98,135.8**	**83,552.8**	**78,215.7**
Battery Production	85.5	682.8	1,491.1	1,491.1	10,338.6	10,338.6
Vehicle Production	13,166.8	12,853.6	12,867.0	12,867.0	10,805.0	10,805.0
Electricity Production	-	-	53,400.5	49,120.3	62,409.2	57,072.1
Gasoline Production	19,381.0	13,011.7	6,573.1	6,573.1	-	-
Vehicle Use	82,696.9	55,604.9	28,084.4	28,084.4	-	-
**SO2**	**52.1**	**106.2**	**97.9**	**92.5**	**240.9**	**233.2**
Battery Production	1.1	51.4	13.8	13.8	153.4	153.4
Vehicle Production	42.9	49.6	49.8	49.8	49.3	49.3
Electricity Production	-	-	31.8	26.4	38.2	30.5
Gasoline Production	7.6	5.0	2.4	2.4	-	-
Vehicle Use	0.6	0.3	0.1	0.1	-	-
**PM2.5**	**6.9**	**6.5**	**8.8**	**8.0**	**9.6**	**8.6**
Battery Production	0.1	0.3	0.3	0.3	2.2	2.2
Vehicle Production	2.4	2.4	2.4	2.4	2.0	2.0
Electricity Production	-	-	3.7	2.8	4.3	3.2
Gasoline Production	2.1	1.5	0.7	0.7	-	-
Vehicle Use	2.3	2.3	1.7	1.7	1.2	1.2
**VOC**	**148.3**	**133.1**	**108.1**	**107.3**	**79.1**	**78.2**
Battery Production	0.0	0.2	0.3	0.3	1.9	1.9
Vehicle Production	71.9	72.1	72.1	72.1	70.5	70.5
Electricity Production			5.9	5.0	6.7	5.9
Gasoline Production	31.4	21.1	10.5	10.5	-	-
Vehicle Use	44.9	39.6	19.3	19.3	-	-
**NOx**	**51.4**	**40.9**	**67.3**	**60.2**	**67.8**	**60.8**
Battery Production	0.1	0.7	1.7	1.7	13.1	13.1
Vehicle Production	13.1	12.6	12.6	12.6	10.2	10.2
Electricity Production	-	-	39.2	32.1	44.5	37.5
Gasoline Production	26.7	17.9	9.1	9.1	-	-
Vehicle Use	11.5	9.7	4.8	4.8	-	-
**CO**	**488.7**	**483.6**	**280.8**	**272.9**	**63.2**	**55.1**
Battery Production	0.0	0.0	0.2	0.2	1.1	1.1
Vehicle Production	41.4	41.9	42.2	42.2	36.1	36.1
Electricity Production	-	-	23.0	15.1	26.0	17.8
Gasoline Production	16.7	11.2	5.6	5.6	-	-
Vehicle Use	430.5	430.5	209.9	209.9	-	-

Table 6: Consequential emissions in 2025 (lb per vehicle lifetime) across powertrains for six types of pollutants, assuming 10% of the light-duty passenger car fleet in PJM's service area is replaced with new cars. "ICEV" denotes a conventional (internal combustion) vehicle, "HEV" denotes a standard hybrid electric vehicle, "PHEV20" denotes a plug-in hybrid electric vehicle with a battery range of 20 miles, and "BEV300" denotes a pure battery electric with a battery range of 300 miles. "CC" indicates that battery charging is optimally scheduled by PJM to minimize system operations costs, and "UC" indicates that battery charging is uncontrolled (i.e., initiated by the vehicle owner as soon as they complete their daily driving and arrive home).

			**PHEV20**		**BEV300**	
	**ICEV**	**HEV**	**UC**	**CC**	**UC**	**CC**
**GHGs**	**115,330.3**	**82,152.9**	**88,082.4**	**91,228.2**	**65,806.0**	**70,685.2**
Battery Production	85.5	682.8	1,491.1	1,491.1	10,338.6	10,338.6
Vehicle Production	13,166.8	12,853.6	12,867.0	12,867.0	10,805.0	10,805.0
Electricity Production	-	-	39,066.9	42,212.6	44,662.4	49,541.6
Gasoline Production	19,381.0	13,011.7	6,573.1	6,573.1	-	-
Vehicle Use	82,696.9	55,604.9	28,084.4	28,084.4	-	-
**SO2**	**52.1**	**106.2**	**91.4**	**90.2**	**229.5**	**236.0**
Battery Production	1.1	51.4	13.8	13.8	153.4	153.4
Vehicle Production	42.9	49.6	49.8	49.8	49.3	49.3
Electricity Production	-	-	25.3	24.1	26.8	33.3
Gasoline Production	7.6	5.0	2.4	2.4	-	-
Vehicle Use	0.6	0.3	0.1	0.1	-	-
**PM2.5**	**6.9**	**6.5**	**7.1**	**7.4**	**7.5**	**8.1**
Battery Production	0.1	0.3	0.3	0.3	2.2	2.2
Vehicle Production	2.4	2.4	2.4	2.4	2.0	2.0
Electricity Production	-	-	2.0	2.2	2.2	2.8
Gasoline Production	2.1	1.5	0.7	0.7	-	-
Vehicle Use	2.3	2.3	1.7	1.7	1.2	1.2
**VOC**	**148.3**	**133.1**	**107.0**	**106.9**	**77.8**	**77.8**
Battery Production	0.0	0.2	0.3	0.3	1.9	1.9
Vehicle Production	71.9	72.1	72.1	72.1	70.5	70.5
Electricity Production	-	-	4.8	4.7	5.5	5.4
Gasoline Production	31.4	21.1	10.5	10.5	-	-
Vehicle Use	44.9	39.6	19.3	19.3	-	-
**NOx**	**51.4**	**40.9**	**54.9**	**54.3**	**53.6**	**54.1**
Battery Production	0.1	0.7	1.7	1.7	13.1	13.1
Vehicle Production	13.1	12.6	12.6	12.6	10.2	10.2
Electricity Production	-	-	26.8	26.2	30.3	30.8
Gasoline Production	26.7	17.9	9.1	9.1	-	-
Vehicle Use	11.5	9.7	4.8	4.8	-	-
**CO**	**488.7**	**483.6**	**271.4**	**270.8**	**52.8**	**52.4**
Battery Production	0.0	0.0	0.2	0.2	1.1	1.1
Vehicle Production	41.4	41.9	42.2	42.2	36.1	36.1
Electricity Production	-	-	13.6	13.0	15.5	15.2
Gasoline Production	16.7	11.2	5.6	5.6	-	-
Vehicle Use	430.5	430.5	209.9	209.9	-	-

Table 7: Consequential emissions (lb per vehicle lifetime) in 2035, assuming 10% renewable energy generation, across powertrains for six types of pollutants, assuming 10% of the light-duty passenger car fleet in PJM's service area is replaced with new cars. "ICEV" denotes a conventional (internal combustion) vehicle, "HEV" denotes a standard hybrid electric vehicle, "PHEV20" denotes a plug-in hybrid electric vehicle with a battery range of 20 miles, and "BEV300" denotes a pure battery electric with a battery range of 300 miles. "CC" indicates that battery charging is optimally scheduled by PJM to minimize system operations costs, and "UC" indicates that battery charging is uncontrolled (i.e., initiated by the vehicle owner as soon as they complete their daily driving and arrive home).

			**PHEV20**		**BEV300**	
	**ICEV**	**HEV**	**UC**	**CC**	**UC**	**CC**
**GHGs**	**115,330.3**	**82,152.9**	**91,583.1**	**92,921.3**	**70,114.2**	**73,283.8**
Battery Production	85.5	682.8	1,491.1	1,491.1	10,338.6	10,338.6
Vehicle Production	13,166.8	12,853.6	12,867.0	12,867.0	10,805.0	10,805.0
Electricity Production	-	-	42,567.5	43,905.7	48,970.6	52,140.2
Gasoline Production	19,381.0	13,011.7	6,573.1	6,573.1	-	-
Vehicle Use	82,696.9	55,604.9	28,084.4	28,084.4	-	-
**SO2**	**52.1**	**106.2**	**85.2**	**94.9**	**224.0**	**236.4**
Battery Production	1.1	51.4	13.8	13.8	153.4	153.4
Vehicle Production	42.9	49.6	49.8	49.8	49.3	49.3
Electricity Production	-	-	19.1	28.8	21.3	33.7
Gasoline Production	7.6	5.0	2.4	2.4	-	-
Vehicle Use	0.6	0.3	0.1	0.1	-	-
**PM2.5**	**6.9**	**6.5**	**7.4**	**8.3**	**7.8**	**9.2**
Battery Production	0.1	0.3	0.3	0.3	2.2	2.2
Vehicle Production	2.4	2.4	2.4	2.4	2.0	2.0
Electricity Production	-	-	2.2	3.2	2.5	3.8
Gasoline Production	2.1	1.5	0.7	0.7	-	-
Vehicle Use	2.3	2.3	1.7	1.7	1.2	1.2
**VOC**	**148.3**	**133.1**	**107.2**	**106.9**	**78.1**	**77.9**
Battery Production	0.0	0.2	0.3	0.3	1.9	1.9
Vehicle Production	71.9	72.1	72.1	72.1	70.5	70.5
Electricity Production	-	-	4.9	4.7	5.7	5.5
Gasoline Production	31.4	21.1	10.5	10.5	-	-
Vehicle Use	44.9	39.6	19.3	19.3	-	-
**NOx**	**51.4**	**40.9**	**60.1**	**53.6**	**58.1**	**54.2**
Battery Production	0.1	0.7	1.7	1.7	13.1	13.1
Vehicle Production	13.1	12.6	12.6	12.6	10.2	10.2
Electricity Production	-	-	31.9	25.5	34.8	30.9
Gasoline Production	26.7	17.9	9.1	9.1	-	-
Vehicle Use	11.5	9.7	4.8	4.8	-	-
**CO**	**488.7**	**483.6**	**272.4**	**276.5**	**54.3**	**59.4**
Battery Production	-	0.0	0.2	0.2	1.1	1.1
Vehicle Production	41.4	41.9	42.2	42.2	36.1	36.1
Electricity Production	-	-	14.6	18.7	17.0	22.2
Gasoline Production	16.7	11.2	5.6	5.6	-	-
Vehicle Use	430.5	430.5	209.9	209.9	-	-

Table 8: Consequential emissions (lb per vehicle lifetime) in 2035, assuming 22% renewable energy generation, across powertrains for six types of pollutants, assuming 10% of the light-duty passenger car fleet in PJM's service area is replaced with new cars. "ICEV" denotes a conventional (internal combustion) vehicle, "HEV" denotes a standard hybrid electric vehicle, "PHEV20" denotes a plug-in hybrid electric vehicle with a battery range of 20 miles, and "BEV300" denotes a pure battery electric with a battery range of 300 miles. "CC" indicates that battery charging is optimally scheduled by PJM to minimize system operations costs, and "UC" indicates that battery charging is uncontrolled (i.e., initiated by the vehicle owner as soon as they complete their daily driving and arrive home).

			**PHEV20**		**BEV300**	
	**ICEV**	**HEV**	**UC**	**CC**	**UC**	**CC**
**GHGs**	**115,330.3**	**82,152.9**	**93,591.6**	**95,871.7**	**71,561.9**	**76,347.9**
Battery Production	85.5	682.8	1,491.1	1,491.1	10,338.6	10,338.6
Vehicle Production	13,166.8	12,853.6	12,867.0	12,867.0	10,805.0	10,805.0
Electricity Production	-	-	44,576.0	46,856.1	50,418.3	55,204.3
Gasoline Production	19,381.0	13,011.7	6,573.1	6,573.1	-	-
Vehicle Use	82,696.9	55,604.9	28,084.4	28,084.4	-	-
**SO2**	**52.1**	**106.2**	**94.5**	**108.3**	**233.5**	**253.2**
Battery Production	1.1	51.4	13.8	13.8	153.4	153.4
Vehicle Production	42.9	49.6	49.8	49.8	49.3	49.3
Electricity Production	-	-	28.4	42.1	30.7	50.5
Gasoline Production	7.6	5.0	2.4	2.4	-	-
Vehicle Use	0.6	0.3	0.1	0.1	-	-
**PM2.5**	**6.9**	**6.5**	**8.0**	**8.7**	**8.3**	**9.8**
Battery Production	0.1	0.3	0.3	0.3	2.2	2.2
Vehicle Production	2.4	2.4	2.4	2.4	2.0	2.0
Electricity Production	-	-	2.9	3.6	3.0	4.4
Gasoline Production	2.1	1.5	0.7	0.7	-	-
Vehicle Use	2.3	2.3	1.7	1.7	1.2	1.2
**VOC**	**148.3**	**133.1**	**107.2**	**106.8**	**78.0**	**77.6**
Battery Production	0.0	0.2	0.3	0.3	1.9	1.9
Vehicle Production	71.9	72.1	72.1	72.1	70.5	70.5
Electricity Production	-	-	4.9	4.5	5.6	5.3
Gasoline Production	31.4	21.1	10.5	10.5	-	-
Vehicle Use	44.9	39.6	19.3	19.3	-	-
**NOx**	**51.4**	**40.9**	**57.0**	**58.5**	**54.9**	**59.3**
Battery Production	0.1	0.7	1.7	1.7	13.1	13.1
Vehicle Production	13.1	12.6	12.6	12.6	10.2	10.2
Electricity Production	-	-	28.9	30.4	31.6	36.0
Gasoline Production	26.7	17.9	9.1	9.1	-	-
Vehicle Use	11.5	9.7	4.8	4.8	-	-
**CO**	**488.7**	**483.6**	**273.2**	**273.8**	**53.7**	**57.1**
Battery Production	-	0.0	0.2	0.2	1.1	1.1
Vehicle Production	41.4	41.9	42.2	42.2	36.1	36.1
Electricity Production	-	-	15.4	16.0	16.4	19.8
Gasoline Production	16.7	11.2	5.6	5.6	-	-
Vehicle Use	430.5	430.5	209.9	209.9	-	-

Figure 16: Sources of new electricity generation induced in our 2019—with reserve requirements (base case) and without (sensitivity case)—when 10% of the light-duty passenger car fleet in PJM's service area is replaced with a new BEV with a range of 300 miles. ("Other", including generation such as nuclear, hydroelectric, landfill gas, and petroleum coke, increased by a negligible amount.

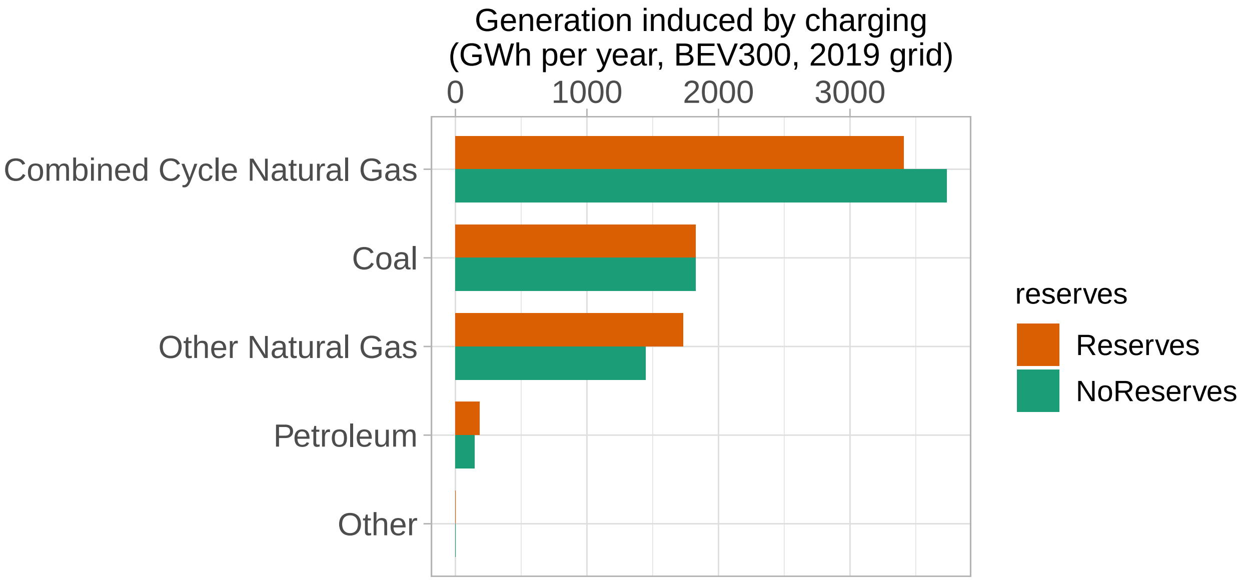

Table 9: Summary of changes to empirically observed coal fleet average emissions factors (EFs) (weighted by generating capacity) in PJM from 2010 to 2019.

	2010 average EFs			
	Total	Retired by 2019	Still active in 2019	2019 average EFs
NOX	2.04	2.95	1.47	1.87
SO2	9.84	12.89	7.94	2.36
N2O	0.04	0.04	0.03	0.04
GHGs	2116.95	2189.83	2071.45	2198.48
CO	0.27	0.31	0.25	0.50
NH3	0.00	0.01	0.00	0.00
PM10	0.84	1.08	0.70	0.19
PM25	0.73	0.93	0.60	0.16
VOC	0.03	0.04	0.02	0.02
% of capacity with a scrubber (NEEDS)	66.8%	46.5%	79.5%	82.5%

Table 10: Sets, decision variables, and input parameters

Label	Type	Description
$\mathcal{I}$	Set	Generating units
$\mathcal{K}$	Set	Storage units
$\mathcal{R}$	Set	Transmission constraint regions (TCRs)
$\mathcal{T}$	Set	Timesteps
$\mathcal{V}$	Set	Plug-in electric vehicle schedule groups
$c_{v,r,t}^{\mathrm{VEH}}$	Variable	Charge level (MWh) of vehicle schedule group v in TCR r at time t
$c_{k,t}^{\mathrm{STR}}$	Variable	Charge level (MWh) of storage unit k at time t
$p_{i,t}^{\mathrm{GEN}}$	Variable	Power generated by unit i at time t
$p_{k,t}^{\mathrm{STR}}$	Variable	Power discharged (+) or charged (−) by storage unit k at time t
$p_{r,r',t}^{\mathrm{RR}}$	Variable	Power imported (+) or exported (−) from TCR r to TCR $r'(\neq r)$ at time t
$p_{v,r,t}^{\mathrm{VR}}$	Variable	Power charged (−) by vehicle schedule group v in TCR r at time t
$s_{i,t}^{\mathrm{STARTUP}}$	Variable	Slack variable for startup cost
$u_{i,t}$	Variable	Binary variable that is equal to 1 if unit i is online in period t and 0 otherwise
$A_{v,t}$	Parameter	Availability of vehicle schedule group v to charge at time t (%)
B_v	Parameter	Battery capacity (MWh) of vehicle grouping v
c_k^{STRMAX}	Parameter	Max charge level (MWh) of storage unit k
C_i^{VARGEN}	Parameter	Variable cost of generating unit i
C_i^{STARTGEN}	Parameter	Startup cost of generating unit i
C_i^{FIXGEN}	Parameter	Fixed cost when generating unit i is online
$P_{r,t}$	Parameter	Power demand in TCR r at time t
$p_{r,r',t}^{\mathrm{RRMAX}}$	Parameter	Max power flow on interface (r, r') at time t
$M_{v,t}$	Parameter	Miles traveled per car in vehicle schedule group v at time t
$N_{v,r}$	Parameter	Number of vehicles in schedule group v in TCR r
p_i^{GENMIN}	Parameter	Min generation from unit i at time t
p_i^{GENMAX}	Parameter	Max generation from unit i at time t
p_k^{STRMIN}	Parameter	Min discharge rate from storage unit k
p_k^{STRMAX}	Parameter	Max discharge rate from storage unit k
R_i	Parameter	Ramp rate limit of unit i
R_v^{MAXCHG}	Parameter	Max charge rate (MW) of vehicle schedule group v
T_i^{UPMIN}	Parameter	Minimum uptime of unit i
T_i^{DTMIN}	Parameter	Minimum downtime of unit i
T_i^{UPSTART}	Parameter	Number of timesteps unit i has been online at initial timestep $t = 1$
T_i^{DTSTART}	Parameter	Number of timesteps unit i has been offline at initial timestep $t = 1$
T_v^{DEPART}	Parameter	Timestep at which vehicle schedule group v departs home
η_v^{CHG}	Parameter	Charging efficiency (%) of vehicle schedule group v
η_k^{STR}	Parameter	Efficiency (%) of storage unit k
η_v^{VEH}	Parameter	Driving efficiency (MWh/mile) of vehicle schedule group v

Table 11: Optimization problem formulation.

$$\text{minimize} \sum_{t\in\mathcal{T}} \sum_{r\in\mathcal{R}} \sum_{i\in I_r} \left(C_i^{\text{VARGEN}} p_{i,t}^{\text{GEN}} + s_{i,t}^{\text{STARTUP}} + C_i^{\text{FIXGEN}} u_{i,t} \right)$$

Subject to

**System constraints:**			
$P_{r,t} = \sum_{i\in\mathcal{I}_r} p_{i,t}^{\text{GEN}} + \sum_{k\in\mathcal{K}_r} p_{i,t}^{\text{STR}} + \sum_{r'\in\mathcal{R}\neq r} p_{r,r',t}^{\text{RR}} + \sum_{v\in\mathcal{V}_r} p_{v,r,t}^{\text{VR}}$	$\forall r \in \mathcal{R}, t \in \mathcal{T}$	Demand must equal supply	(1)
$-p_{r,r',t}^{\text{RRMAX}} \leq p_{r,r',t}^{\text{RR}} \leq p_{r,r',t}^{\text{RRMAX}}$	$\forall r \in \mathcal{R}, r' \in \mathcal{R} \neq r, t \in \mathcal{T}$	Inter-region power flow limits	(2)
**Storage constraints:**			
$c_{k,t+1}^{\text{STR}} = c_{k,t}^{\text{STR}} - p_{k,t}^{\text{STR}} / \eta^{STR}$	$\forall k \in \mathcal{K}, \forall t \in \mathcal{T}$	Storage state of charge	(3)
$0 \leq c_{k,t}^{\text{STR}} \leq c_k^{\text{STRMAX}}$	$\forall k \in \mathcal{K}, \forall t \in \mathcal{T}$	Storage unit capacity	(4)
$p_k^{\text{STRMIN}} \leq p_{k,t}^{\text{STR}} \leq p_k^{\text{STRMAX}}$	$\forall k \in \mathcal{K}, \forall t \in \mathcal{T}$	Max charge and discharge of storage units	(5)
**Generator constraints:**			
$s_{i,t}^{\text{STARTUP}} \geq \left(u_{i,t} - u_{i,t-1}\right) C_i^{\text{STARTGEN}}$	$\forall i \in \mathcal{I}, \forall t \in \mathcal{T}$	Slack variable reflects startup decision	(6)
$s_{i,t}^{\text{STARTUP}} \geq 0$	$\forall i \in \mathcal{I}, \forall t \in \mathcal{T}$	Slack variable is nonnegative	(7)
$p_i^{\text{GENMIN}} u_{i,t} \leq p_{i,t} \leq p_i^{\text{GENMAX}} u_{i,t}$	$\forall i \in \mathcal{I}, \forall t \in \mathcal{T}$	Min and max output of online generators	(8)
$p_{i,t}^{\text{GEN}} \leq p_{i,t-1}^{\text{GEN}} + R_i u_{i,t-1} + p_i^{\text{GENMIN}} \left(u_{i,t} - u_{i,t-1}\right)$	$\forall i \in \mathcal{I}, \forall t \in \mathcal{T}$	Ramp rate limit	(9)
$p_{i,t-1}^{\text{GEN}} \leq p_{i,t}^{\text{GEN}} + R_i u_{i,t} + p_i^{\text{GENMIN}} \left(u_{i,t-1} - u_{i,t}\right)$	$\forall i \in \mathcal{I}, \forall t \in \mathcal{T}$	Ramp rate limit	(10)
$\sum_{t=2}^{T_i^{\text{UPMIN}} - T_i^{\text{UPSTART}}} \left(1 - u_{i,t}\right) = 0$	$\forall i \in \mathcal{I}$	Minimum uptime (first timesteps)	(11)
$\sum_{t'=t}^{t+T_i^{\text{UPMIN}}-1} u_{i,t'} \geq T_i^{\text{UPMIN}} \left(u_{i,t} - u_{i,t-1}\right)$	$\forall i \in \mathcal{I}$, $\forall t : T_i^{\text{UPMIN}} - T_i^{\text{UPSTART}} + 1 \leq t \leq \lvert\mathcal{T}\rvert - T_i^{\text{UPMIN}} + 1$	Minimum uptime (middle timesteps)	(12)
$\sum_{t=t}^{T} u_{i,t'} \geq (T-t) \left(u_{i,t} - u_{i,t-1}\right)$	$\forall i \in \mathcal{I}$, $\forall t \in \lvert\mathcal{T}\rvert - T_i^{\text{UPMIN}} + 2 \ldots$	Minimum uptime (final timesteps)	(13)
$\sum_{t=2}^{T_i^{\text{DTMIN}} - T_i^{\text{DTSTART}}} u_{i,t} = 0$	$\forall i \in \mathcal{I}$	Minimum downtime (first timesteps)	(14)
$\sum_{t'=t}^{t+T_i^{\text{DTMIN}}-1} 1 - u_{i,t'} \geq T_i^{\text{DTMIN}} \left(u_{i,t-1} - u_{i,t}\right)$	$\forall i \in \mathcal{I}$, $\forall t : T_i^{\text{DTSTART}} + 1 \leq t \leq \lvert\mathcal{T}\rvert - T_i^{\text{DTSTART}} + 1$	Minimum downtime (middle timesteps)	(15)
$\sum_{t'=t}^{T} 1 - u_{i,t'} \geq \left(\lvert\mathcal{T}\rvert - t\right) \left(u_{i,t-1} - u_{i,t}\right)$	$\forall i \in \mathcal{I}$, $\forall t : \lvert\mathcal{T}\rvert - T_i^{\text{DTSTART}} + 2 \leq t \leq \lvert\mathcal{T}\rvert$	Minimum downtime (final timesteps)	(16)
**PEV charge constraints:**			
$c_{v,r,t+1}^{\text{VEH}} = c_{v,r,t}^{\text{CHG}} - p_{v,r,t}^{\text{VR}} \eta_v^{\text{VEH}} - N_{v,r} M_{v,t} \eta_v^{\text{VEH}}$	$\forall v \in \mathcal{V}, \forall r \in \mathcal{R}, \forall t \in \mathcal{T}$	PEV state of charge	(17)
$-N_{v,r} A_{v,t} R_v^{\text{MAXCHG}} \leq p_{v,r,t}^{\text{VR}} \leq 0$	$\forall v \in \mathcal{V}, \forall r \in \mathcal{R}, \forall t \in \mathcal{T}$	PEV charge rate limit	(18)
$0 \leq c_{v,r,t}^{\text{VEH}} \leq N_{v,r} B_v$	$\forall v \in \mathcal{V}, \forall r \in \mathcal{R}, \forall t \in \mathcal{T}$	PEV battery capacity	(19)
$c_{v,r,t}^{\text{VEH}} = N_{v,r} B_v$	$\forall v \in \mathcal{V}, \forall r \in \mathcal{R}, \forall t = T_v^{\text{DEPART}}$	PEVs fully charged when departing home	(20)

Table 12: Summary of Year 2035 generator fleet scenarios. Each scenario's renewable energy assumption is defined in terms of total demand (which is held constant across scenarios). For dispatchable generating units, assumptions are defined in terms of installed capacity.

RE output assumption	Installed capacity scenarios		Installed capacity values (GW)			
	Coal	Natural Gas	Coal	Natural Gas	Nuclear	Other
10% of demand	EIA860 schedule	EIA860 schedule	34	93	31	6
	EIA860 schedule minus 25% remaining	EIA860 schedule	26	93	31	6
		EIA860 schedule + backfill of extra 25% coal retired	26	100	31	6
	EIA860 schedule minus 50% remaining	EIA860 schedule	17	93	31	6
		EIA860 schedule + backfill of extra 50% coal retired	17	109	31	6
22% of demand	EIA860 schedule	EIA860 schedule	34	93	31	6
	EIA860 schedule minus 25% remaining	EIA860 schedule	26	93	31	6
		EIA860 schedule + backfill of extra 25% coal retired	26	100	31	6
	EIA860 schedule minus 50% remaining	EIA860 schedule	17	93	31	6
		EIA860 schedule + backfill of extra 50% coal retired	17	109	31	6

porate Average Fuel Economy Policy and Greenhouse Gas Emissions Standards. *Environmental Science & Technology* **2016**, *50*, 2165–2174, PMID: 26867100.

(6) Jenn, A.; Azevedo, I. L.; Michalek, J. J. Alternative-fuel-vehicle policy interactions increase U.S. greenhouse gas emissions. *Transportation Research Part A: Policy and Practice* **2019**, *124*, 396–407.

(7) U.S. Energy Information Administration, Petroleum Refineries. 2022; `https://atlas.eia.gov/datasets/eia::petroleum-refineries-1/`, Date last accessed: 6 Dec 2023.

(8) U.S. Energy Information Administration, Natural Gas Processing Plants. 2022; `https://atlas.eia.gov/datasets/eia::natural-gas-processing-plants`, Date last accessed: 6 Dec 2023.

(9) U.S. Energy Information Administration, Coal Mines. 2022; `https://atlas.eia.gov/datasets/eia::coal-mines`, Date last accessed: 6 Dec 2023.

(10) Bruchon, M. B.; Michalek, J. J.; Azevedo, I. L. Effects of Air Emission Externalities on Optimal Ridesourcing Fleet Electrification and Operations. *Environmental Science & Technology* **2021**, *55*, 3188–3200, PMID: 33601882.

(11) Forsythe, C.; Jha, A.; Michalek, J.; Whitefoot, K. Externalities of Policy-Induced Scrappage: The Case of Automotive Regulations. *NBER Working Paper Series* **2022**, 1–69.

(12) Weis, A.; Jaramillo, P.; Michalek, J. Consequential life cycle air emissions externalities for plug-in electric vehicles in the PJM interconnection. *Environmental Research Letters* **2016**, *11*, 024009.
